# Optimized melanin production from marine-associated *Bacillus* sp. EGY7 as a sustainable precursor of carbon dots for biomedical applications

**DOI:** 10.1186/s40643-026-01100-w

**Published:** 2026-07-27

**Authors:** Hadeer Ali, Labiba El-Kohordagui, Ahmed Hussein, Sherif Hammad, Nefertiti El‑Nikhely, Nehad Noby

**Affiliations:** 1 Biotechnology Department, Institute of Graduate Studies and Research, Alexandria University, Alexandria, Egypt; 2 Department of Pharmaceutics, Faculty of Pharmacy, Alexandria University, Alexandria, Egypt; 3 Pharmaceutical Chemistry Department, Faculty of Pharmacy, Capital University (formerly Helwan University), Cairo, 11795, Egypt; 4 Medicinal chemistry Department, Faculty of Pharmacy, Egypt-Japan University of Science and Technology (E-JUST), New Borg-EL Arab City, 21934 Alexandria, Egypt; 5 Molecular Biotechnology Program, Alamein International University, New Alamein, Egypt

**Keywords:** Marine-associated *Bacillus* sp, Box–Behnken design, Microbial melanin, Melanin carbon dots, Cytocompatibility, Selective anticancer activity, In silico bioactivity

## Abstract

Melanin, a multifunctional biopigment with photoprotective, antioxidant, and therapeutic properties, is limited by scarce sustainable sources and poor aqueous solubility. In this study, melanin production from a newly isolated marine-associated *Bacillus* sp. EGY7 was optimized using a Box–Behnken design, achieving a yield of 0.208 g/L. The microbial melanin was subsequently hydrothermally converted into water-dispersible melanin-derived carbon dots (MCDs) with quasi-spherical morphology, particle size of 9–15 nm, and a quantum yield of 5.05 ± 0.31%. The MCDs exhibit strong UV absorption, tunable and often excitation-dependent photoluminescence (PL), high photostability, and environment-sensitive fluorescence governed by their core–shell structure and surface functional groups. MCDs exhibited moderate antioxidant activity and high cytocompatibility, maintaining > 70% viability in human skin fibroblasts. Compared with native melanin, MCDs demonstrated enhanced selective cytotoxicity against breast cancer cells (IC_50_: 0.25 mg/mL for MCF-7 and 0.68 mg/mL for MDA-MB-231), with selectivity indices reaching 19.6 and superior inhibition of long-term colony formation. Mechanistic investigations suggested that their anticancer activity is mediated by reactive oxygen species (ROS)-induced apoptosis coupled with efficient nanoscale cellular uptake. Complementary in silico analyses predicted interactions with multiple cancer-related targets involved in apoptosis, oxidative stress regulation, and cell cycle control, supporting their theragnostic potential. Overall, these findings establish *Bacillus* sp. EGY7 as a renewable melanin source and highlight MCDs as a sustainable multifunctional platform for selective anticancer and bioimaging applications.

**Graphical abstract:**

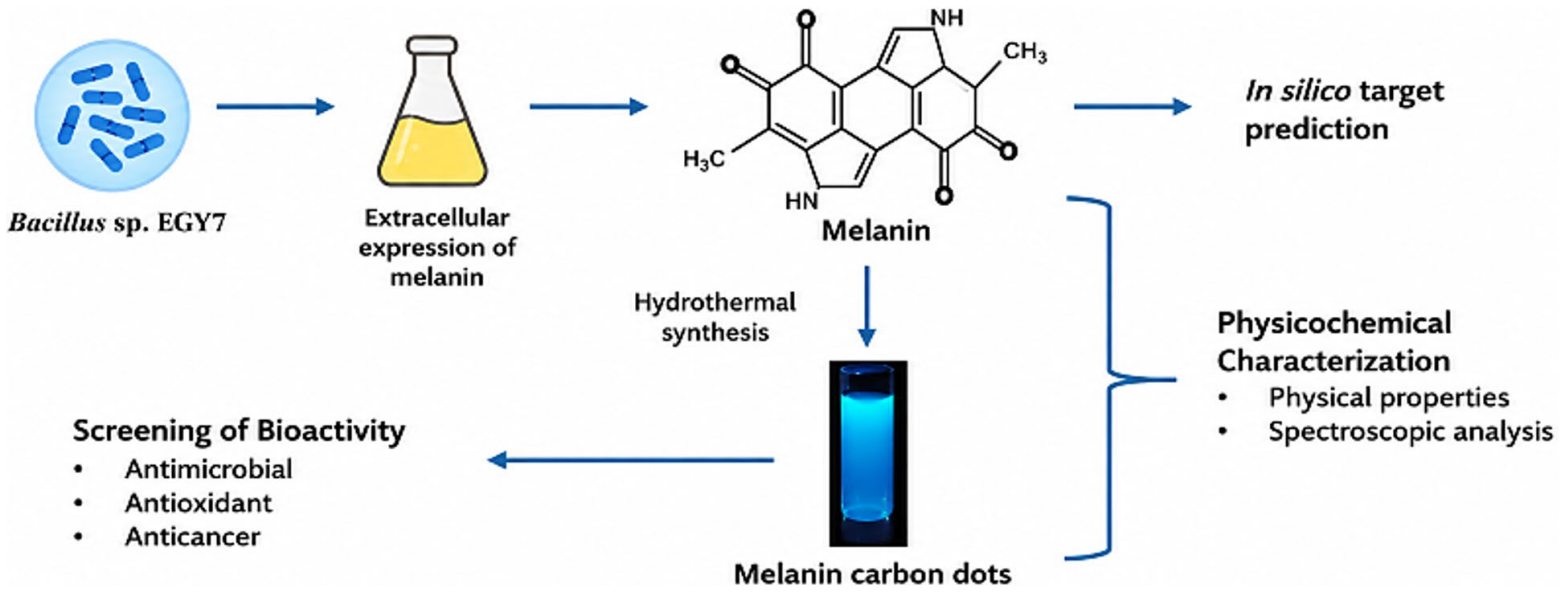

**Supplementary Information:**

The online version contains supplementary material available at 10.1186/s40643-026-01100-w.

## Introduction

Melanin, a dark heterogeneous biopolymer produced across the biosphere by bacteria, fungi, plants, and animals, plays key roles in pigmentation and protection from ultraviolet (UV) radiation and oxidative stress (Cordero And Casadevall 2020). In humans, it shields cells from UV damage and determines pigmentation in skin, hair, and eyes. Beyond coloration, melanin offers antioxidant activity, scavenges reactive oxygen species (ROS), chelates metal ions, and aids immune regulation, enhancing cellular resilience to environmental and oxidative stressors (Quintus 2025; Pandey et al. 2026).

Melanin pigments arise from the oxidative polymerization of phenolic and indolic precursors, yielding structural classes such as eumelanin, pheomelanin, and pyomelanin, each characterized by a conjugated, redox-active framework (Solano 2014). Eumelanin, the predominant black-to-brown pigment derived from L-tyrosine oxidation, is the most extensively studied owing to its broad-spectrum light absorption, free-radical scavenging capacity, redox activity, and metal-chelating properties (Singh et al. 2025). Melanin pigments also exhibit antibacterial, antioxidant, and anticancer activities (Polapally et al. 2022) with reported effects against several cancer types, including breast cancer (Marcovici et al. 2025; Abdulla et al. 2026; Li et al. 2020). Collectively, these properties position melanin as a versatile bioinspired material for applications in biomedicine, cosmetics, environmental remediation, and biotechnology.

Nevertheless, the practical application of melanin is limited by its unsustainable sourcing, high production costs, and poor solubility in water and most organic solvents. Chemical synthesis often requires harsh reagents and energy-intensive processes, leading to toxicity issues and product heterogeneity (Cao et al. 2021; Pralea et al. 2019) while extraction from natural sources such as cuttlefish ink (Song et al. 2021) or plants (Aghajanyan et al. 2024) is labor-intensive and yields relatively low quantities. These limitations have driven growing interest in microbial fermentation as a scalable and more sustainable production strategy, particularly under optimized cultivation conditions.

A wide range of microorganisms, including fungi (Suthar and Singh 2026) and bacteria such as *Bacillus* (An et al. 2024; Marín-Sanhueza et al., 2022), *Streptomyces* (Kordjazi et al. 2024; Uasoontornnopa et al. 2025; Cimini et al. 2026), and *Pseudomonas* (Khorshid And Odaa 2025; Eskandari And Etemadifar 2021) have been identified as efficient melanin producers. In addition to terrestrial sources, marine microorganisms derived from diverse ecological niches also represent a promising but underexplored reservoir of melanin-producing species. These marine microbes often synthesize structurally unique melanins adapted to extreme environmental conditions, further highlighting their biotechnological potential (Ghattavi et al. 2022; Attia et al. 2025; Wilson et al. 2024)**.** Microbial melanin biosynthesis is generally mediated by tyrosinase-, laccase-, or polyketide synthase (PKS)-dependent pathways which differ in precursor usage and enzymatic steps (Pavan et al. 2020). In fungi and actinobacteria such as *Streptomyces,* melanin is commonly produced via the PKS-driven 1,8-dihydroxynaphthalene (DHN) pathway, whereas in melanogenic *Bacillus* species, L-tyrosine is converted to L-DOPA intracellularly and subsequently oxidized and polymerized extracellularly by enzymes such as laccases to form eumelanin (Hullo et al. 2001; Drewnowska et al. 2015). This diversity of mechanisms enables efficient pigment production across different ecological niches.

A key barrier to melanin’s biomedical use is its poor dispersibility and solubility in water and most organic solvents, hindering formulation and biomedical translation. Physical methods like ultrasonication (Suarez-Vergel et al. 2026) and chemical modifications such as sulfonation (Cuba et al. 2021) improve dispersibility, though the latter raises safety issues. Encapsulation within liposomes (Kim And Lee 2022; Casula et al. 2025), hydrogels (Wu et al. 2021) or polymer matrices (Roy et al. 2020; Wu et al. 2021) enables stable platforms for drug delivery, photoprotection, and antioxidant therapy.

In parallel, nanotechnology has broadly enabled the engineering of diverse material systems with tailored physicochemical properties for a wide range of biomedical applications (Mousavi et al. 2026; Ger et al. 2025; Islam et al. 2026). Within this broader landscape, melanin-based nanostructures have emerged as a particularly promising class of nanomaterials. Recent studies have highlighted their growing relevance in nanomedicine, owing to their tunable physicochemical and biological properties, which can be leveraged for both therapeutic and diagnostic applications (Park et al. 2019; Marcovici et al. 2022, 2025; Unnikrishnan et al. 2025). Melanin-like nanoparticles (MNPs; 20–200 nm), synthesized via oxidative polymerization of catecholamines like dopamine or 5,6-dihydroxyindole, mimic natural melanin and excel in antioxidant/anticancer activities, including photothermal drug release (Xu et al. 2025; Wu et al. 2025; Menichetti et al. 2025). Synthetic melanin nanoparticles (SMNPs), from polydopamine or analogs, provide precise size/surface control for targeted delivery (Tian et al. 2022). Melanin nanodots (MNDs; < 10 nm), formed by neutralizing alkaline melanin solutions with acids (e.g., HCl), offer amorphous structure, NIR absorption, and ROS scavenging for anti-inflammatory, photothermal, and radical protection uses (Sun et al. 2025; Li et al. 2024; Shi et al. 2021). Recently, ultrasmall disc-like nanoparticles (2–6 nm) assemble into tunable spherical supraparticles (30–100 nm), enabling hierarchical control (Madhu et al. 2026).

Within the broad filed of nanomaterials, carbon dots (CDs) have emerged as a particularly remarkable branch in the field of biological sciences. Carbon dots (CDs) are synthesized through either top-down or bottom-up methods. Bottom-up method, which involves hydrothermal or microwave-assisted pyrolysis is highly favoured due to its eco-friendly nature and low toxicity, in addition, this pathway preserves the intrinsic properties of the starting material (Usman And Cheng 2024). Carbon dots (CDs) are widely utilized in biomedical applications for cellular bioimaging, selective biosensing of biomarkers in addition to targeted drug delivery (Wang et al. 2022). Furthermore, they serve a vital role in cancer treatment by combining diagnostic imaging with photothermal or photodynamic therapies to destroy malignant cells (Lan et al. 2018). Recently, melanin carbonaceous dots, quasi-spherical, graphitic nanostructures (< 10–30 nm), have emerged via high-temperature carbonization of melanin precursors using hydrothermal or microwave-assisted methods (Xiao et al. 2017; Hu et al. 2016) or isolation from natural sources like *Aplysia dactylomela* ink (Khataminejad et al. 2025). Unlike MNPs, melanin carbonaceous dots feature a quantum-confined carbon core and abundant surface groups, enabling tunable fluorescence, high aqueous dispersibility, and versatile chemistry for bioimaging, fluorescence sensing, targeted drug delivery, and theragnostics (Liu et al. 2025a).

Despite their promising properties, microbial melanin-derived carbon dots (MCDs) remain insufficiently explored for biomedical applications, underscoring the need for sustainable and scalable production strategies. In this study, we report a newly isolated marine-associated *Bacillus* sp. strain EGY7 as a melanin-producing microorganism. Adapted to harsh marine conditions such as high salinity, UV exposure, and temperature fluctuations, the strain demonstrates robust growth characteristics that may be advantageous for scalable production. We further show its environmentally friendly conversion into carbon dots. The resulting material exhibits dispersibility, intrinsic fluorescence, and biocompatibility, suggesting potential for biomedical applications. This work integrates microbial melanin biosynthesis with green nanomaterial synthesis, providing a bio-based platform for functional carbon nanomaterials.

## Materials and methods

Peptone, yeast extract, ammonium sulphate ((NH_4_)_2_SO_4_), potassium mono-hydrogen phosphate (K_2_HPO_4_.2H_2_O), sodium thiosulphate (Na_2_S_2_O_3_), ferrous sulphate (Fe_2_SO_4_), copper sulphate (CuSO_4_.5H_2_O), glycerol and agar were obtained from Biolife Co., Italy. L-tyrosine was purchased from Lanxess AG, Germany. Universal primers for 16S rRNA were synthesized by Willowfort, UK. Standard Master Mix 2X was purchased from Promega, USA. Methanol, absolute ethanol, N-methyl-2-pyrrolidone, dimethyl sulphoxide (DMSO), chloroform, Tween 20, acetone, urea, and sodium hydroxide (NaOH), were obtained from Piochem (Piochem Laboratory Chemicals), Egypt.

### Production of bacterial melanin

#### Isolation of melanin-producing bacterial strains from seawater samples

Samples of seawater collected from El-Alamein and other beaches in Alexandria, Egypt were first activated in 50 mL of a basal nutritional medium designated as Medium 1 (Table S1) containing (per liter): 20 g peptone, 1 g yeast extract, 0.5 g (NH_4_)_2_SO_4_, 1 g K_2_HPO_4_, 0.08 g Na_2_S_2_O_3_, and 0.5 g Fe_2_SO_4_.5H_2_O; pH 8.0 and incubated at 30°C with shaking at 150 rpm for 48 h. The activated cultures were then spread onto plates of the same medium and incubated for a further 48 h. The purified isolates were preserved in 20% glycerol at − 80°C.

#### Identification of the melanin-producing strain

The potential melanin-producing isolate was identified based on tyrosinase activity, morphological characteristics, and molecular analysis. Tyrosinase activity was measured in the cell-free lysate using L-tyrosine as the substrate. For lysate preparation, freshly activated colonies were inoculated into 50 mL of basal Medium 1 supplemented with 0.6 g/L L-tyrosine and incubated at 30 °C with shaking at 200 rpm for 48 h. Cells were then harvested by centrifugation at 2000 × g for 15 min, washed with 50 mM Tris–HCl buffer (pH 8.0), and disrupted by sonication at 18 kHz for 5 min with 1-min intervals on ice. The cell-free lysate was obtained by centrifugation at 4472 × g for 10 min and subsequently used for the enzymatic assay.

Tyrosinase activity was determined by monitoring the oxidation of L-DOPA to the red chromophore dopachrome, following the method described by El-Naggar and El-Ewasy (2017). Briefly, the reaction mixture consisted of 2.0 mL of 10 mM L-DOPA in 50 mM Tris–HCl buffer (pH 8.0), 0.5 mL of cell-free lysate, and 0.5 mL of the same buffer, and was incubated at 30 °C for 10 min. Dopachrome formation was measured spectrophotometrically at 475 nm. The blank contained 0.5 mL of cell-free lysate, 0.5 mL of Tris–HCl buffer, and 1.0 mL of deionized water. One unit (U) of tyrosinase activity was defined as the amount of enzyme required to produce 1 µmol of dopachrome per min under the assay conditions at 30°C (Yang and Wu 2006).

Colony morphology and microscopic features were examined and the isolate was further identified molecularly via 16S rRNA gene sequencing following the method of Eden et al. (1991). Genomic DNA was extracted using the ZYMO DNA extraction kit (D3024) according to the manufacturer’s instructions. The universal primers 27F (5′-AGAGTTTGATCMTGGCTCAG-3′) and 1492R (5′-TACGGYTACCTTGTTACGACTT-3′) (Heuer et al. 1997) were employed for amplification of the 16S rRNA gene by polymerase chain reaction (PCR). The amplified product was purified and sequenced using an automated fluorescent DNA Sequencer. The resulting partial nucleotide sequence was analyzed using the BLASTn tool (NCBI), and a phylogenetic tree was constructed with MEGA 11 software to determine the taxonomic affiliation of the isolate.

#### Optimization of melanin production

The basal nutritional medium for melanin production (Medium 1) was optimized using a sequential approach. A one-variable-at-a-time (OVAT) method as a first optimization step was employed to identify the components that significantly influence pigment yield. Accordingly, five modified formulations of Medium 1 were prepared. The detailed compositions of all six media are listed in Table S1 in the supplementary file. All experiments were conducted in triplicate in 250 mL Erlenmeyer flasks containing 50 mL working volume of medium, inoculated with 5% (v/v) preculture, and incubated at 30°C with shaking at 200 rpm for 7 days. Melanin concentration was determined quantitatively by measuring optical density at 280 nm (El-Naggar And El-Ewasy 2017) and the yield was expressed as g L⁻^1^. Statistical significance was determined at *p* ≤ 0.05 using the overlapping standard error bars approach described by Cumming et al. (Cumming et al. 2007).

The OVAT analysis yielded a partially optimized medium with the following composition in (g/L): 20 g peptone, 1 g yeast extract, 0.5 g (NH4)_2_SO_4_,1g K_2_HPO_4_, 0.1 g L-Tyrosine, 0.04 g CuSO_4_ g, 0.5 g FeSO_4_.5H_2_O. Although OVAT analysis has improved pigment production compared to Medium 1, it doesn’t account for possible interactions among variables. Therefore, the concentrations of the three most significant factors identified, L-tyrosine (X₁), CuSO_4_ (X_2_), and K_2_HPO_4_ (X_3_), were further optimized using response surface methodology (RSM) based on a Box–Behnken design (BBD) to maximize melanin yield (Gidwani And Vyas 2016).

Each independent variable was tested in three coded levels, denoted as -1 (low level), 0 (center level), and + 1 (high level), through a full design matrix of 15 experimental trials (Table S2 in the supplementary file**)**. Melanin yield, expressed in g/L was selected as the dependent variable (response). The coded actual values of the three independent variables are listed in Table 1. Each trial was performed in 250 mL Erlenmeyer flasks containing 50 mL medium, inoculated with 5% v/v of a 48 h preculture (~ 1.0 × 10^6^ CFU), and incubated at 30°C with shaking at 200 rpm for 7 days. The three independent variables were represented by Eq. 1:

1 $$\begin{aligned} {\text{Y }} = & {\text{ }}\beta 0{\text{ }} + {\text{ }}\beta {\text{1 }}\left( {{\mathrm{X1}}} \right){\text{ }} + {\text{ }}\beta {\mathrm{2}}\left( {{\mathrm{X2}}} \right){\text{ }} + {\text{ }}\beta {\mathrm{3}}\left( {{\mathrm{X3}}} \right){\text{ }} \\ & + {\text{ }}\beta {\mathrm{12}}\left( {{\mathrm{X1X2}}} \right){\text{ }} + {\text{ }}\beta {\mathrm{13}}\left( {{\mathrm{X1X3}}} \right) \\ & + {\text{ }}\beta {\text{ 23}}\left( {{\mathrm{X2X3}}} \right){\text{ }} + {\text{ }}\beta {\text{ 11}}\left( {{\mathrm{X1}}} \right){\text{ 2 }} \\ & + {\text{ }}\beta {\mathrm{22}}\left( {{\mathrm{X2}}} \right){\text{ 2 }} + {\text{ }}\beta {\text{ 33}}\left( {{\mathrm{X3}}} \right) \\ \end{aligned}$$

where Y is the response, β0 is the model intercept, β1, β2, and β3 are linear coefficients, β12, β13, and β23 are cross product coefficients, while β11, β22, and β33 are quadratic coefficients.

**Table 1:** Independent variables of the Box–Behnken design (BBD) with their codes, coded levels, and actual values

Variables	Codes	Coded levels and actual levels		
		− 1	0	1
L-tyrosine % (w/v)	X1	0.05	0.15	0.25
CuSO_4._5H_2_O % (w/v)	X2	0.005	0.015	0.025
K_2_HPO_4_% (w/v)	X3	0.1	0.3	0.5

To validate the model and evaluate its accuracy and stability, a predicted trial obtained from BBD numerical optimization was selected as a checkpoint. This formulation was experimentally verified, and the percent deviation between predicted and observed values was calculated.

### Bacterial melanin extraction, purification and characterization

#### Melanin extraction and purification

Melanin was extracted and purified from the fermentation broth following the method of Eskandari and Etemadifar (2021) with slight modifications. The fermentation medium was centrifuged at 1677 × g for 15 min to separate the cell pellets. The supernatant was collected, and the pH was adjusted to 1.0 using concentrated HCl to hydrolyze associated proteins and carbohydrates. The mixture was then incubated in a water bath at 50 °C for 1 h. After centrifugation, the melanin pigment was recovered as a dark brown precipitate. The crude pigment was further purified by sequential extraction with chloroform and ethyl acetate to remove bound carbohydrates, lipids, and proteins to prevent melanoidins formation. The precipitate was washed repeatedly with ultrapure water to pH 7.0 and lyophilized. Melanin solubility was assessed in ultrapure water, methanol, ethanol, N-methyl-2-pyrrolidone, DMSO, chloroform, acetone, and 1% Tween 20.

#### Melanin characterization

##### Microscopic examination

The surface morphology of the purified melanin was examined using scanning electron microscopy (SEM; Quanta 200 FEG, FEI Company, Netherlands) operated at an accelerating voltage of 20 kV. Approximately 5 mg of the dried melanin powder was evenly spread as a thin layer on carbon tape and coated with a thin layer of gold by sputtering under high vacuum prior to imaging.

##### Spectroscopic analyses

A set of spectroscopic techniques was employed to characterize the purified bacterial melanin:

*Ultraviolet–visible (UV–Vis) spectroscopy* The UV–Vis spectrum of the purified pigment dissolved in 0.1 M NaOH was recorded using a Thermo Fisher Scientific spectrophotometer (Madison, WI, USA) over the range of 200–700 nm, with 0.1 M NaOH as the blank.

*Fourier-transform infrared (FT-IR) analysis* Dried melanin powder (2–5 mg) was placed in a salt cuvette and analyzed using an FT-IR spectrometer (Perkin Elmer, Germany) over the range of 4000–450 cm^−1^.

*NMR spectroscopy* ^1^H and ^13^C NMR spectra were obtained at room temperature using a Delta II NMR spectrometer (500 MHz, JOEL, USA). Approximately 15 mg of dried melanin was dissolved in 10 mL of DMSO-d_6_ prior to measurement.

*Electrospray Ionization Mass Spectrometry (ESI–MS)* The molecular weight distribution of the purified melanin pigment was analyzed using a mass spectrometer (XEVO TQD triple quadrupole, Waters Corporation, Milford, MA, USA). Electrospray ionization mass spectrometry (ESI–MS) was performed in both positive- and negative-ion acquisition modes using an ACQUITY UPLC system equipped with a BEH C18 column (2.1 × 50 mm, 1.7 µm particle size). Chromatographic separation was carried out at a flow rate of 0.2 mL min⁻^1^. The solvent system consisted of water and acetonitrile (1:1, v/v), each containing 0.1% formic acid. Samples were directly infused into the ion source without prior chromatographic separation.

### Synthesis and characterization of bacterial melanin carbon dots (MCDs)

#### Synthesis of bacterial MCDs

Synthesis of MCDs was carried out through hydrothermal treatment according to a previously reported method applied on synthetic melanin pigment with slight modification (Hu et al. 2016). Briefly, a 5 mg/mL solution of purified bacterial melanin in 0.1 M NaOH was subjected to hydrothermal treatment at 200°C for 36 h. After cooling, the mixture was centrifuged at 7000 rpm (8050 × g) for 10 min to remove unreacted material. The resulting supernatant was further purified by filtration through a 0.22 µm membrane and subsequently dialyzed against deionized water until the pH stabilized at 7.0–7.5.

#### Physicochemical characterization of MCDs

*Physical properties* The morphology of the prepared MCDs was examined using TEM (JEOL 2100 PLUS, 200 kV, Japan) and their particle size was determined using the TEM software. In addition, the zeta potential of the MCDs was measured with a Malvern Zetasizer Nano ZS (Malvern Instruments, UK).

#### Spectroscopic analyses

*UV–Vis spectroscopy*The optical absorption profile of the prepared MCDs was recorded using a UV–Vis spectrophotometer (Madison, WI, USA) in the wavelength range of 200–600 nm, with deionized water (DIW) as the blank.

*Photoluminescence (PL) properties* PL measurements of the MCDs were performed using a helium–cadmium laser beam at 330 nm excitation. Emission spectra were recorded across different wavelengths with a fluorescence spectrophotometer (PerkinElmer LS 55, USA). The fluorescence quantum yield (QY) of the MCDs was determined relative to quinine sulfate according to Eq. 2:

2 $$Q_{s} = Q_{r} \left( {\frac{{m_{s} }}{{m_{r} }}} \right)\left( {\frac{{n_{s} }}{{n_{r} }}} \right)^{2}$$

where: ${Q}_{s}$= quantum yield of the sample (MCDs), ${Q}_{r}$= quantum yield of the reference (quinine sulfate, typically 0.54 in 0.1 M H_2_SO_4_, ${m}_{s},{m}_{r}$= slopes of the plots of integrated fluorescence intensity vs. absorbance for sample and reference, respectively, ${n}_{s},{n}_{r}$= refractive indices of the solvents used for sample and reference. This comparative method ensures accurate determination of QY by correcting for differences in absorbance and solvent refractive index.

*Stability* The pH stability of the prepared MCD dispersions (5 mg mL⁻^1^) was investigated in Milli-Q water (pH 7.0), 50 mM acetate buffer (pH 5.0), and 50 mM Tris–HCl buffer (pH 9.0) at 4 °C. Photostability was evaluated by exposing the dispersions to continuous illumination from an electric lamp in a stability chamber at ambient temperature (~ 20 °C), and changes in their PL intensity were monitored over time. PL measurements were recorded immediately after preparation (t = 0) and subsequently under identical instrumental conditions, including the same excitation wavelength (330 nm) and acquisition parameters. For pH stability studies, PL intensity was measured after 72 h of incubation. Photostability was assessed after 72 h of continuous light exposure. Fluorescence retention was calculated according to Eq. 3:

3 $${\text{Retention }}\left( \% \right) \, = \, \left( {{\mathrm{I}}_{{\mathrm{t}}} /{\mathrm{I}}_{{0}} } \right) \, \times { 1}00$$

where I₀ is the fluorescence intensity at 0 h and Iₜ is the fluorescence intensity at the emission maximum at the corresponding incubation time.

To monitor potential changes in the optical properties and colloidal behavior of the MCDs during incubation, UV–Vis absorption spectra were also recorded at the designated time points.

*Fourier-transform infrared (FTIR) analysis* spectra were obtained on a PerkinElmer spectrometer (Germany) in the range of 450–4000 cm^−1^.

*Raman spectroscopy* The Raman spectrum of the MCDs was recorded using a Raman microscope (SENTERRA, Bruker, Germany) with an excitation wavelength of 785 nm.

*Energy-dispersive X-ray spectroscopy (EDX)* The elemental composition of MCDs was analyzed by EDX coupled to a scanning electron microscope (JEOL 1400 Plus SEM). Spectra were acquired at a voltage of 120 kV over the range 0–20 keV, and elemental weight and atomic percentages were quantified.

*X-ray photoelectron spectroscopy (XPS)* XPS analysis was performed to investigate the surface chemistry and elemental composition of the MCDs. The purified MCD dispersion was dried in a vacuum oven at 37 °C overnight until a constant weight was achieved. The resulting dried material was gently ground into a fine powder and pressed into pellets prior to analysis. XPS measurements were conducted using an X-ray photoelectron spectrometer (Thermo Fisher Scientific, USA).

## Biological characterization of MCDs

### In silico analysis and prediction of biological activity

The canonical SMILES of melanin was retrieved from PubChem with the ID 6325610 (CC1=C2C3=C(C4=CNC5=C(C(=O)C(= O)C(=C45)C3=CN2)C)C(= O)C1=O) and was used for the following in silico analyses. First, SWISS AMDE online tools stipulated its pharmacokinetics generating a radar diagram representing the main 6 features affecting the bioavailability of a drug (i.e. size, polarity, insolubility, insaturation, flexibility and lipophilicity). The colored area represents suitability of physicochemical properties for oral bioavailability. This was confirmed by MolSoft tool (https://molsoft.com/mprop/) to predict drug-likeness and molecular property prediction (Krisnamurti et al. 2025).

Subsequently, the spectrum of biological activity was predicted using PASS online prediction tool where melanin was screened for various biological activities. For each biological activity, the probability to be active (Pa) and the probability to be inactive (Pi) were calculated, Having a higher Pa than Pi indicates a potential to possess the respective biological activity (Filimonov et al. 2014).

#### Antimicrobial activity

The antimicrobial activity of the prepared MCDs was evaluated against *E. coli* ATCC 25922, *S. aureus* ATCC 29213, and *C. albicans* ATCC 90028 using the agar well diffusion method (Perez 1990)*.* Each microbial strain was cultivated separately on solidified agar plates. Wells of 6 mm diameter were cut into the agar using a sterile cork borer and filled with 100 µL of MCDs at varying concentrations (6.0, 3.0, 1.0, 0.5, and 0.25 mg/mL). Sterile water served as the negative control. To ensure uniform diffusion of the test solutions, the plates were first incubated at 4°C for 1 h, followed by incubation at 37°C overnight. Zones of inhibition surrounding the wells containing MCDs were measured and compared with those of the control wells.

#### Antioxidant effect

The antioxidant efficiency of MCDs was assessed using the radical scavenging ability test (RSA) using 2,2-diphenyl-1-picrylhydrazyl (DPPH) (Sachdev And Gopinath 2015). One milliliter of 70 µM DPPH solution was mixed with 1 mL of MCDs dispersions of different concentrations. The sample volume was replaced with ethanol in the control sample. The mixtures were incubated in the dark for 1 h, after which the absorbance was measured at 517 nm. The radical scavenging activity at each MCDs concentration was calculated using Eq. 4:

4 $${\text{E }}\% \, = \, \left[ {\left( {{\text{A control}} - {\text{ A sample}}} \right) \, /{\text{ A control}}} \right] \, *{1}00$$

where E % represents the percentage of radical scavenging efficiency.

#### Cytocompatibility

The cytocompatibility of MCDs was evaluated using normal human skin fibroblast (HSF) cells obtained from American Type Culture Collection (ATCC), via Nawah Scientific, Egypt. Cells were maintained at 37°C in a humidified incubator with 5% CO_2_ and cultured in Dulbecco’s Modified Eagle Medium (DMEM) supplemented with fetal bovine serum and antibiotics. After seeding into 96‑well plates, cells were exposed the following day to freshly prepared MCDs at concentrations ranging from 50 to 800 µg/mL, prepared by 2-fold serial dilutions, for assessment using the MTT assay (van de Loosdrecht et al. 1991). Following 24 h of incubation, MTT reagent was added and incubated for 3 h. The resulting formazan crystals were solubilized in DMSO, and absorbance was measured at 570 nm using a microplate reader. Data were analyzed with GraphPad Prism software (USA). Cell viability (%) was calculated according to Eq. 5:

5 $$\% \mathrm{Viability}=\left( {\frac{{{\text{OD treated cells }} - {\text{ OD media blank}}}}{{{\text{OD vehicle control }} - {\text{ OD media blank}}}}} \right) \times 100$$

where OD vehicle control denotes the mean absorbance of cells treated with deionized water, OD treated cells represents the mean absorbance of cells exposed to MCDs at each concentration, and OD media blank corresponds to the mean absorbance of wells containing complete medium only, without cells.

#### Anticancer activity

*Cytotoxicity on cancer cells using MTT assay* The cytotoxic potential of MCDs, compared with melanin, was evaluated in the triple-negative breast cancer cell line MDA-MB-231 and the human breast adenocarcinoma cell line MCF-7 using the MTT assay. The procedure was performed as described above following treatment with melanin at varying concentrations (prepared in DMSO/0.1 M NaOH) and MCDs (dispersed in water) within the range of 50 to 800 µg/mL. The respective vehicle solutions served as controls. After treatment, IC_50_ values were determined for each sample in each cell line and compared with the IC_50_ values obtained for HSF cells using Eq. 5. The selectivity index (SI) was then calculated according to Eq. 6:

6 $$\text{Selectivity index}=\frac {\text{{IC50 on normal cells}}}{\text{IC50 on cancer cells}}$$

*Colony forming assay* The colony-forming assay was performed as previously described by Franken et al. (Franken et al. 2006). MCF-7 and MDA-MB-231 cells were seeded in 12‑well plates at a density of 250 cells/well and allowed to adhere for 24 h without treatment. Subsequently, cells were exposed to melanin or MCDs at a concentration of 0.2 mg/mL. Treatment medium was refreshed every two days for 7 days until optimal colony formation was achieved. At the end of the incubation period, cells were washed with PBS, fixed, and stained with 6% glutaraldehyde and 0.5% crystal violet for 30 min. Colonies were quantified using ImageJ software with the ColonyArea plugin. All experiments were performed in triplicate, and results are expressed as mean ± SD.

### Molecular target prediction for melanin

*Prediction of molecular targets* The SMILES representation of melanin, (CC1=C2C3=C(C4=CNC5=C(C(= O)C(= O)C(= C45)C3=CN2)C)C(= O)C1=O), was used as input for target prediction. Melanin was screened using the SwissTargetPrediction tool (http://swisstargetprediction.ch/) to identify potential molecular targets in human cells.

*Gene set enrichment analysis* Pathway analysis by MSigDB Hallmark 2020 was performed for the targets to identify major pathways affected by melanin. Predicted targets were sorted by scores and represented as clusters. The online tool enrichR was used for the enrichment analysis of gene ontology (GO). The GO terms enriched in the gene dataset were represented based on their relevance score.

*Quantitative RT-PCR* Cells were treated with either melanin (M) or MCDs for 24h, after which cells were lysed and their mRNA isolated by TRIzol reagent as recommended by the manufacturer. Once the quality and purity of the mRNA were checked by Nanodrop, the expression of CDK1, CCND1, and CCNE1 was measured by qPCR using HERA SYBR Green RT-qPCR kit, Willowfort, UK. The qPCR was performed using SaCycler 96 real time PCR system, Sacace Biotechnologies, Italy, and the following primers were used: HPRT sense 5′- TGACACTGGCAAAACAAT-3′, HPRT antisense 5′- GGTCCTTTTCACCAGCAA-3′, CDK1 sense 5′-CAATTTCTGAATCCCCATGG-3′, CDK1 antisense 5′- GTAGTAACACTCTGGTACAG-3′; CCND1 sense 5′- GCTGCGAAGTGGAAACCATC-3′, CCND1 antisense 5′- CCTCCTTCTGCACACATTTGAA-3′; and CCNE1 sense 5′- AGAGGAAGGCAAACGTGACC-3′ CCNE1 antisense 5′- TATTGTCCCAAGGCTGGCTC-3′. HPRT was used as house keeping gene.

### Statistical analysis

Data were analyzed using GraphPad Prism version 6 (GraphPad Software Inc., CA, USA). Results are presented as mean ± SD, with statistical significance set at *p* < 0.05. Minitab Statistical Software (version 22) was used to generate the Box–Behnken design. Multiple linear regression analysis was conducted using Microsoft Excel 2010 to evaluate model significance based on p-values. The maximal predicted response and optimal levels of the independent variables were determined using the Solver add-in in Microsoft Excel 2010. The combined effects of the three variables were visualized using a 3D response surface plot constructed with STATISTICA software (version 7.0).

## Results and discussion

In this study, MCDs were synthesized from the newly isolated marine-associated *Bacillus* sp. strain EGY7 and comprehensively characterized. By integrating microbial fermentation with nanoengineering techniques, CDs exhibiting enhanced physicochemical properties relative to native melanin were developed, offering a sustainable and scalable pathway for producing nanomaterials with promising biological applications.

### Isolation and identification of melanin-producing bacterial isolate

**Fig. 1:**
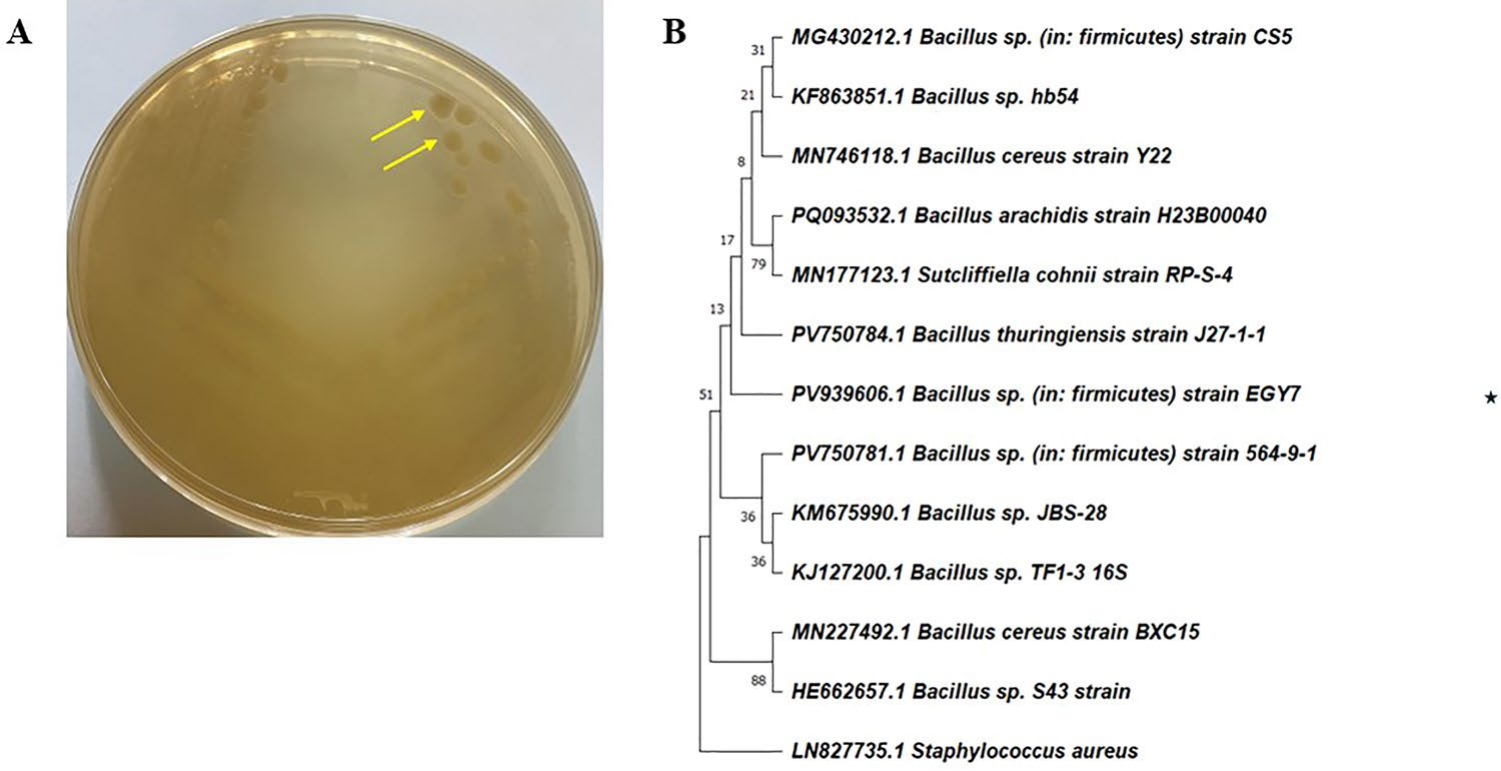
Identification of the melanin-producing isolate. **A** Colonies of *Bacillus* sp. EGY7 grown on basal Medium 1 supplemented with L-tyrosine, showing dark brown pigmentation (indicated by yellow arrows). **B** Phylogenetic relatedness between EGY7 strain sequence and closely related bacterial strains using Neighbour joining method. Accession number is indicated for each genus. Bootstrap values are represented as a percentage of 500 replicates

Screening of marine bacterial isolates from Alexandria seawater identified a single strain from Alamein beach with positive tyrosinase activity on L-tyrosine–supplemented medium, indicating melanin-producing potential. The isolate formed smooth, rounded, dark brown colonies (Fig. 1A), consistent with tyrosinase-mediated oxidation of polyphenolic substrates (Nikolaivits et al. 2018)**.** Phylogenetic analysis of the 16S rRNA sequence (Fig. 1B) and BLAST search confirmed close affiliation with the genus *Bacillus*. The strain was designated *Bacillus* sp. EGY7 and deposited in GenBank (accession No. PV939606.1). Given the ubiquitous distribution of the genus *Bacillus* across diverse environments, the growth resilience of strain EGY7 was evaluated on marine agar medium (Microxpress^®^, India). The strain exhibited growth comparable to that observed on the formulated medium, indicating its halotolerant nature. Based on its marine origin and tolerance to elevated salinity, EGY7 was classified as a marine-associated strain, in agreement with previously reported marine-derived *Bacillus* isolates exhibiting comparable physiological traits (Lu et al. 2026; Wang et al. 2020)**.** Notably, *Bacillus* species are increasingly recognized as robust microbial platforms for melanin production owing to their metabolic versatility and scalability (Ghadge et al. 2020; An et al. 2024), underscoring the relevance of *Bacillus* sp. EGY7 as a renewable source of melanin.

The phylogenetic relationship between strain EGY7 and previously reported melanin-producing *Bacillus* strains (KF585035.1, MZ298610.1, KP006648.1, and OM967420.1) was analyzed using MEGA11 software. The resulting phylogenetic tree revealed that EGY7 clustered with the *Bacillus weihenstephanensis* clade with 100% bootstrap support based on 500 replicates (Supplementary file, Fig. S1). Previous studies have shown that melanin biosynthesis in *B. weihenstephanensis* is associated with a multicopper oxidase/laccase-mediated pathway (Drewnowska et al. 2015). Therefore, the close phylogenetic affiliation of EGY7 with *B. weihenstephanensis* suggests that melanin production in EGY7 may proceed through a similar laccase-dependent biosynthetic mechanism.

### *Bacillus* sp. EGY7-produced melanin

#### Preliminary identification

**Fig. 2:**
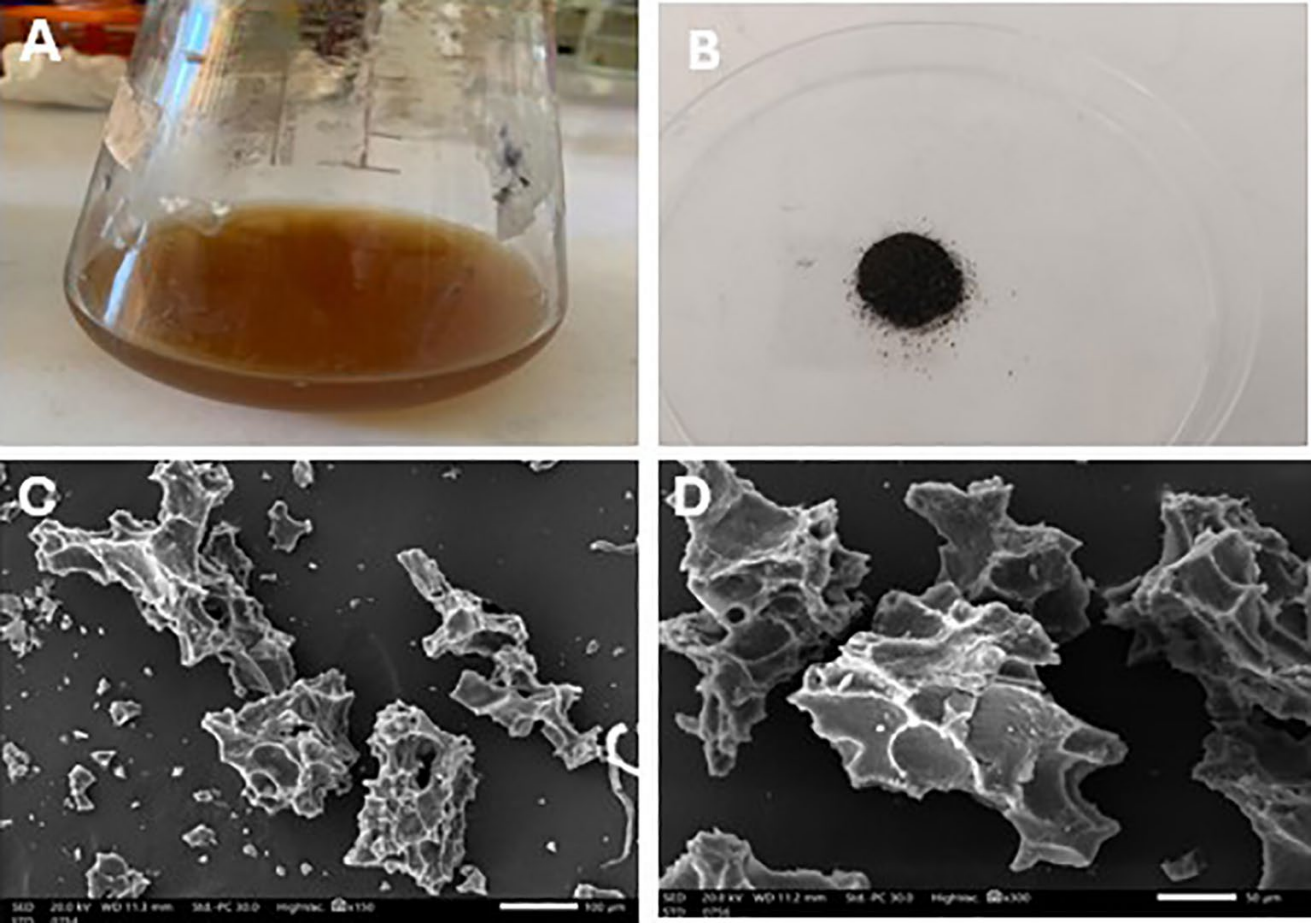
Morphological characterization of melanin from *Bacillus* sp. EGY7 **A** Accumulation of extracellular pigment during prolonged incubation; **B** Morphology of purified melanin after drying; **C** and **D** SE micrographs of purified melanin at 100 × and 300 × , respectively

*Bacillus* sp. EGY7 produced melanin extracellularly, as evidenced by the progressive darkening of the culture medium with prolonged incubation (Fig. 2A). The purified pigment was recovered as a fine black powder upon drying (Fig. 2B). A typical preliminary step in pigment identification is the qualitative determination of the solubility profile of melanin, which is known to be insoluble in water and polar and nonpolar solvents but soluble in aqueous alkali solutions. In this study, *Bacillus* sp. EGY7-produced melanin exhibited poor solubility in ultrapure water, methanol, ethanol, N-methyl-2-pyrrolidone, DMSO, chloroform, acetone, and 1% Tween 20 micellar solution while it was soluble in 1 M NaOH. This solubility profile is consistent with that reported for melanin from other microbial and natural sources (Kamarudheen et al. 2019; Pralea et al. 2019; El-Zawawy et al. 2024).

#### Morphology by SEM

Scanning electron microscopy (SEM) revealed that melanin produced by *Bacillus* sp. EGY7 formed loosely packed, amorphous aggregates composed of sharp-edged, highly irregular, porous, and non-uniform particles spanning a moderately broad size range (Fig. 2C and D, at 100 × and 300 × , respectively). This distinctive morphology, reflecting heterogeneous polymerization and variable supramolecular stacking of indolic units, aligns with previous descriptions of microbial melanin, including that synthesized by the halophilic black yeast *Hortaea werneckii* (Rani et al. 2013), and *Brevibacillus invocatus* strain IBA (Ammanagi et al. 2021) and *Streptomyces djakartensis* NSS-3 (El-Zawawy et al. 2024).

#### Structural characteristics

**Fig. 3:**
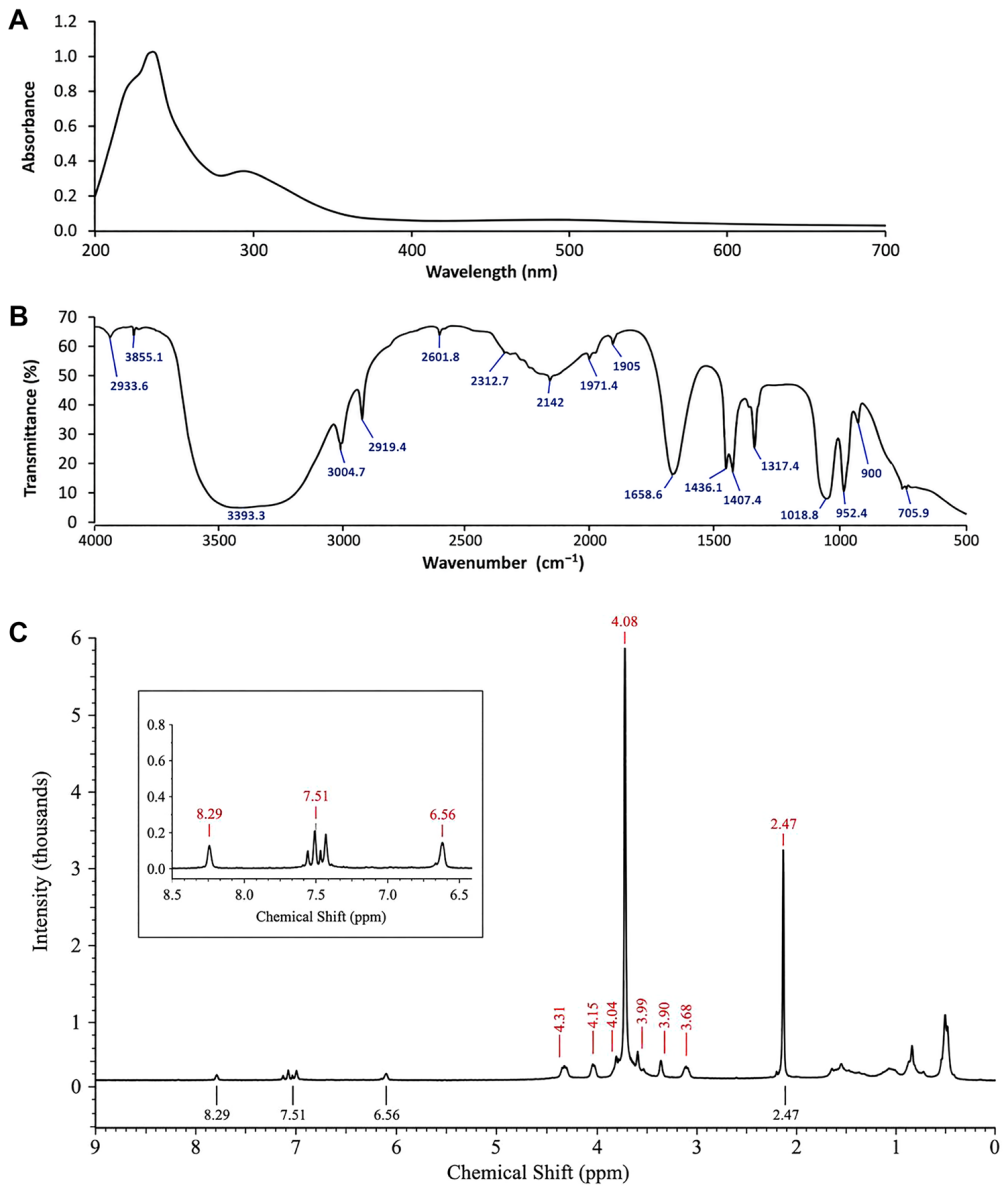
Spectral characterization of purified *Bacillus* sp. EGY7-produced melanin: **A** UV–Vis spectrum, **B** FTIR spectrum and **C** ^1^H NMR spectrum

The UV–Vis spectrum of purified melanin produced by *Bacillus* sp. EGY7 (Fig. 3A) exhibited a prominent absorption peak at 235 nm, attributed to π–π* transitions of aromatic C=C bonds in conjugated polyaromatic systems (Solano 2017). Microbial melanins tends to show broad UV absorption, often peaking in the 200–300 nm range (Song et al. 2023)**.** A broad UV band with a gradual decline into the visible region (400–700 nm) was observed, a spectral signature typical of melanin and melanin-like materials (Noman et al., 2022; El-Zawawy et al. 2024). This profile reflects the abundance of aromatic moieties, mainly indole- and catechol-based units that constitute the polymeric backbone of bacterial melanin (Singh et al. 2021). The absence of peaks at 260–280 nm confirmed the lack of nucleic acid and protein impurities.

The melanin FTIR spectrum (Fig. 3B) displayed characteristic absorption bands consistent with reported melanin profiles (Ghadge et al. 2023). A broad band at 3393–3009 cm^−1^ corresponds to O–H and N–H stretching, indicative of carboxylic acids, phenolic groups, and indole/pyrrole structures. The band at 2919.4 cm⁻^1^ is assigned to aliphatic C–H stretching of –CH_2_ and –CH_3_ groups. A strong band at 1658.4 cm⁻^1^ reflects aromatic C=C and C=N stretching, while peaks at 1435 cm⁻^1^ and 1407 cm^−1^ correspond to aliphatic C–H bending and O–H bending of phenolic/carboxylic groups, respectively (Pralea et al. 2019). The absorption at 1108.8 cm^−1^ is attributed to C–H or C–O in-plane vibrations within aliphatic structures (El-Naggar And El-Ewasy 2017). Collectively, these features confirm the presence of diverse oxygen- and nitrogen-containing groups typical of melanin polymers.

Notably, the FTIR analysis (Fig. 3B) confirmed the effectiveness of the purification process in removing protein and lipid contaminants from the extracted melanin. The absence of the characteristic Amide I (~ 1630 cm^−1^) and Amide II (~ 1540 cm^−1^) absorption bands indicates the lack of detectable protein-derived amide signatures. Since both bands originate from peptide bond vibrations, their absence suggests that the absorption band observed at approximately 1660 cm^−1^ is attributable to the intrinsic conjugated quinone carbonyl groups of melanin rather than residual protein contaminants (Li et al. 2019). Furthermore, the absence of a prominent ester carbonyl absorption band near 1740 cm^−1^ indicates the effective removal of lipid residues (Al‐Kelani And Buthelezi, 2024). Collectively, these FTIR findings confirm the successful purification of the melanin precursor and the resulting MCDs, demonstrating that protein and lipid impurities were effectively eliminated through chloroform/ethyl acetate extraction.

The ^1^H NMR spectrum of purified melanin displays broad and overlapping signals, reflecting its heterogeneous, polymeric nature (Fig. 3C). In the aromatic region (δ 6.5–8.5 ppm), resonances are assigned to protons of pyrrole, indole, and other aromatic moieties, indicative of conjugated π-electron systems typical of melanin derived from phenolic or indolic precursors (Solano 2017; D’Ischia et al. 2015). Signals between δ 3.0–4.2 ppm are due to methylene or methyl protons adjacent to nitrogen or oxygen (–CH_2_–NH–, –CH_2_–OH), likely from amino acid monomers or polar side chains enhancing solubility (Tran-Ly et al. 2020). The heterogeneous aromatic structure, aggregation behavior, and limited proton mobility characteristic of melanin-derived materials may be responsible for the weak or unresolved methyl proton signals. Aliphatic protons at δ 1.0–2.5 ppm are assignable to –CH_3_ and –CH_2_– groups possibly linked to proteinaceous residues or aliphatic linkers, while a signal near δ 2.0 ppm suggests acetate or –CH_2_–C=O groups (Mostert et al. 2012). These features support the classification of *Bacillus*-produced melanin as a eumelanin-like pigment with complex aromatic and aliphatic domains, consistent with previous microbial melanin reports (Ghadge et al. 2023). The carbon framework was further analyzed by ^13^C NMR spectroscopy (Fig. S2 in the supplementary file), which reveals characteristic signals corresponding to diverse carbon environments typical of melanin structures.

Electrospray ionization mass spectrometry (ESI–MS) was employed in both negative and positive ion modes to elucidate the chemical composition and oligomeric nature of *Bacillus* sp. EGY7 melanin. The spectra demonstrate a polydisperse distribution of low- and high-molecular-weight components, consistent with the polymeric character of melanin. In the negative ion mode (ESI^−^) (Fig. S3A in the supplementary file), the base peak at *m/z* 134.9 is consistent with dihydroxyphenyl acetic acid or a related phenolic metabolite, while additional peaks in the range of *m/z* 150–300 suggest fragments or dimeric units derived from catecholamines. A broad distribution of higher *m/z* peaks (300–500) with retention times between 25 and 32 min indicate the presence of polyindolic oligomers or melanin-like polymers. These findings are consistent with previous reports of fungal and bacterial melanin, which similarly exhibit broad mass distributions, structural complexity, and heterogeneous ionization behavior (Pralea et al. 2019). In the positive ion mode (ESI^+^), a major ion at *m/z* 135 corresponds to dopamine or its protonated derivative (Fig. S3B in the supplementary file). At longer retention times (28–33 min), more intense peaks at *m/z* 323, 477, and higher values are detected, likely representing trimeric or tetrameric melanin oligomers generated by oxidative coupling of dopamine or L-DOPA units (D’Ischia et al. 2009). The higher-mass species adhere to the nitrogen rule, suggesting the incorporation of one to three nitrogen atoms characteristic of indolic or pyrrolic structures (Riley 1997). Table 2summarize the significant peaks observed in negative and positive ion modes of ESI–MS, respectively.

**Table 2:** Significant peaks in the negative mode and positive mode of ESI–MS

Peak	RT (min)	The most likely molecular ion[M–H]^−^	Remarks
A . Significant Peaks in the Negative Mode of ESI–MS			
1	0.76	m/z ≈ 134	DOPAC (3,4-dihydroxyphenylacetic acid); 1 N atom (even m/z possible via deprotonation)
2	10.48	m/z ≈ 163–165	L-DOPA (C_9_H_11_NO_4_), M = 197 → [M–H]⁻ = 196 → unlikely match; could be a phenolic acid
3	15.87	m/z ≈ 152	Dopamine quinone dimer or related structure
10–20	25.5–30	m/z ≈ 300–500 +	High MW indole/quinone oligomers (trimeric/tetrameric eumelanin units)

Peak	RT (min)	The Most LikelyMolecular Ion[M–H]^+^	Remarks
B. Significant Peaks in Positive Mode of ESI–MS			
1	1.10	m/z ≈ 135	Dopamine or L-DOPA fragment
3	10.00	m/z ≈ 150–160	C_9_H_9_NO_2_^+^→ consistent with indole-acetic acid
10–33	28–33 +	m/z ≈ 300–700	Polyindole/pyrrole structures, oxidized melanin oligomers

The structural analysis of the obtained melanin pigment supports the concept that *Bacillus* sp. EGY7-produces melanin is a polyfunctional biopolymer composed of carboxylated, aminated, and aromatic subunits generated through oxidative coupling of indolic precursors. The analysis also indicates the presence of a structurally diverse population of ionic species. A trimeric indole-based eumelanin unit with the proposed formula C_24_H_20_N_4_O_6_ (MW ≈ 476 Da) appears to represent the most probable high molecular weight structure. This unit is consistent with three oxidized DOPA or dopamine monomers, bearing carboxylic acid, quinone, and amine functionalities, and interconnected through C2–C3 or C4–C7 linkages of indole rings. Comparable oligomeric structures have been described in the literature for melanin generated via enzymatic or non-enzymatic oxidative polymerization of catecholic precursors, supporting the plausibility of this model (D’Ischia et al. 2015; Ghadge et al. 2023).

#### Optimization of melanin production medium

Given the complexity and variability of melanin biosynthesis, no universally applicable culture medium or cultivation condition has been established. Although several studies have optimized key parameters such as pH, temperature, incubation time, and medium composition (El-Zawawy et al. 2024; Restaino et al. 2024; Zhu et al. 2025; Kraseasintra et al. 2023a), standardized conditions remain elusive. In this study, a two-step systematic optimization of culture medium composition was undertaken to enhance melanin yield from *Bacillus* sp. EGY7.

**Fig. 4:**
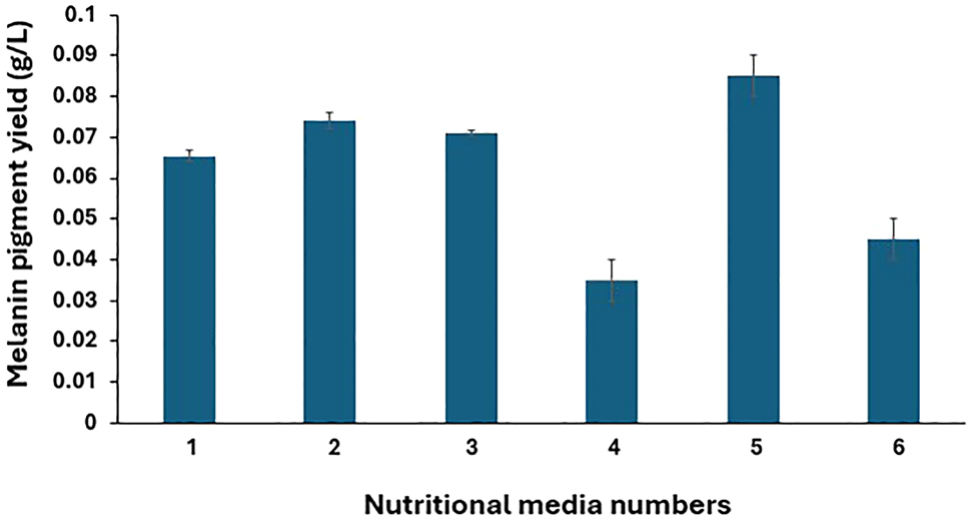
Effect of medium composition on melanin yield. Medium 1 is the basal formulation containing in g/L: peptone (20), yeast extract (1), (NH_4_)_2_SO_4_ (0.5), Na_2_S_2_O_3_ (0.08), K_2_HPO_4_ (1), FeSO_4_.7H_2_O (0.5). Media 2–4 are Medium 1 supplemented with L-tyrosine (0.6), CuSO_4_ (0.04), or MgSO_4_ (0.04), respectively. Media 5 and 6 are Medium 1 lacking Na_2_SO_3_ or K_2_HPO_4_, respectively. Cultures were incubated in triplicate at 30 °C and 200 rpm for 7 days. The significance of differences was assessed using the overlapping rule at *p* ≤ 0.05 (Cumming et al. 2007)

In a preliminary optimization step based on the OVAT approach, modification of the basal medium (Medium 1-Medium 6, Table S1) demonstrated that supplementation with L-tyrosine (Medium 2) or CuSO_4_ (Medium 3), as well as omission of Na_2_S_2_O_3_ (Medium 5), significantly (*p* < 0.05) enhanced pigment production (Fig. 4). In contrast, supplementation with MgSO_4_ (Medium 4) or omission of K_2_HPO_4_ (Medium 6) resulted in a significant (*p* < 0.05) reduction in pigment yield. Therefore, L-tyrosine, CuSO_4_, and K_2_HPO_4_ were identified as the most influential variables enhancing melanin production by *Bacillus* sp. EGY7 and were selected for subsequent statistical optimization. This finding is consistent with literature reports on eumelanin bioproduction, in which tyrosinase catalyzes the oxidation of L-tyrosine to DOPA, the key precursor of melanin. Tyrosinase is a copper-dependent metalloenzyme containing two Cu atoms at its active site, making both substrate (L-tyrosine) and Cu^2^⁺ (cofactor) availability critical determinants of melanin yield, as demonstrated for *Bacillus licheniformis* MAL (Ragab et al. 2019) and *Brevundimonas* sp. SGJ (Surwase et al. 2013). Moreover, tyrosinase activity is highly pH-dependent, with K_2_HPO_4_ contributing through its buffering capacity. In agreement, supplementation of recombinant *E. coli* cultures with K_2_HPO_4_ stabilized pH and significantly enhanced melanin production (Santos And Stephanopoulos 2008).

The levels of L-tyrosine, CuSO_4_, and K_2_HPO_4_ and their interactions were further optimized by RSM employing a BBD design of 15 runs as summarized in Table S2. Runs were performed in triplicate and the average of melanin yield was used as the response. As shown in Table 3, the observed melanin yields (0.120–0.230 g L⁻^1^) validate the predictive accuracy of the model and align with values reported for other melanin producing microorganisms (0.240–0.310 g/L) (Pandey et al. 2024; Kraseasintra et al. 2023b; Aqlinia et al. 2025). The relatively low yield of melanin recovered from natural sources is influenced by several factors, including the biological source, intracellular or extracellular localization, association with proteins and cell-wall components, extraction pH, solvent type, acid precipitation conditions, temperature, cultivation conditions, and purification procedures (Tang et al. 2025). These factors collectively contribute to the generally low and highly variable recovery yields reported for natural melanin extraction. For example, melanin extracted from *Auricularia heimuer* fermentation broth exhibited a yield of only 0.4042%, while extraction from the fruiting bodies of *Auricularia, auricula-judae* yielded approximately 2.59% (w/w) under optimized conditions, demonstrating the limited recovery typically achievable from some natural biomass (Choi 2021; Ma et al. 2023).

**Table 3:** BBD matrix of three independent variables along with the predicted and experimental responses of pigment production in terms of (g/L)

Trial	Variables			Pigment concentration (g/L)		
	X1	X2	X3	Experimental	Predicted	Residual
1	0	0	0	0.212	0.212	2.78E−17
2	0	0	0	0.212	0.212	2.78E−17
3	0	−1	1	0.146	0.155	− 0.009
4	0	1	−1	0.220	0.210	0.009
5	−1	−1	0	0.128	0.124	0.004
6	0	0	0	0.212	0.212	2.78E−17
7	1	0	−1	0.212	0.217	− 0.005
8	0	1	1	0.230	0.223	0.006
9	0	−1	−1	0.130	0.136	− 0.006
10	−1	0	1	0.236	0.231	0.0053
11	−1	0	−1	0.172	0.170	0.0022
12	1	0	1	0.186	0.188	− 0.0022
13	1	1	0	0.194	0.198	− 0.004
14	1	−1	0	0.120	0.109	0.0115
15	−1	1	0	0.166	0.178	− 0.0115

To predict the optimal levels of the tested variables and the corresponding maximum pigment yield, a second-order polynomial regression equation was constructed based on the experimental responses as shown in Eq. 7.

7 $$\begin{aligned}\mathrm{Y}_{\text{(Pigment yield g/L)}}&=0.212 + 0.00125\text{X1 + 0.0357 X2 + 0,008 X3}\\&\text{ + 0.009 X1X2 - 0.0225 X1X3}\\&\text{ - 0.0015 X2X3 - 0.02 X1X1}\\&\text{ - 0.04 X2X2 + 0.0095 X3X3}\end{aligned}$$

The results of the regression coefficient analysis, summarizing the relative importance of the independent variables and their interactions in influencing melanin production and analysis of variance (ANOVA) summary for the regression model are presented in Tables 4 and 5, respectively. As shown in Table 4, incorporation of CuSO_4_ (X_2_) exerted the greatest positive effect on melanin yield (*p* < 0.001), consistent with its role as an essential tyrosinase cofactor. On the other hand, L-tyrosine (X₁), although the direct substrate for melanin biosynthesis, did not exhibit a statistically significant linear effect (*p* > 0.05) with applied concentrations, which might be explained by the presence of other alternative source of tyrosine like the added peptone. K_2_HPO_4_ (X_3_) showed a modest but non-significant positive effect (*p* = 0.096), possibly reflecting indirect contribution to cellular metabolism.

**Table 4:** Regression coefficients, standard errors, t- and p-values of the Box–Behnken design (BBD) model for melanin production by *Bacillus* sp. EGY7

Term	Coefficients	Standard error	t Stat	*P*-value	Upper 95.0%
Intercept	0.21200	0.00638	33.2170	4.65E−07	0.2284
X1	0.00125	0.00390	0.3198	0.7620	0.0112
X2	0.03575	0.00390	9.1471	0.0003	0.0457
X3	0.00800	0.00390	2.0469	0.0960	0.0180
X1X2	0.00900	0.00552	1.6283	0.1640	0.0232
X1X3	− 0.02250	0.00552	− 4.0708	0.0096	− 0.0082
X2X3	− 0.00150	0.00552	− 0.2714	0.7969	0.0127
X1X1	− 0.0200	0.00575	− 3.4765	0.0177	− 0.0052
X2X2	− 0.0400	0.00575	− 6.9530	0.0009	− 0.0252
X3X3	0.0095	0.00575	1.6513	0.1595	0.0243

**Table 5:** Analysis of variance (ANOVA) summary for the regression model

Parameter	df	SS	MS	F	Significance F
Regression	9	0.02079	0.00231	18.9016	0.0024
Residual	5	0.00061	0.00012		
Total	14	0.02140			

Among interaction terms, the negative effect of X1X3 (*p* < 0.01) indicates that excess phosphate may diminish the benefit of high substrate levels, whereas X1X2 and X2X3 are not significant. Significant negative quadratic terms for X1^2^ and especially X2^2^ confirm that excessive concentrations of substrate or cofactor suppressed pigment production. Overall, these findings highlight the importance of copper supplementation at optimal levels, while underscoring the need for balanced nutrient composition to maximize melanin yield.

The ANOVA results (Table 5) demonstrate that the regression model is highly significant, with an F-value of 18.90 and a corresponding p-value of 0.0024. This indicates that the model reliably explains the variation in melanin production in response to the selected variables. The regression sum of squares (0.02079) accounts for nearly all of the total variation (0.02140), while the residual error is minimal (0.00061), confirming an excellent model fit. The calculated coefficient of determination is high (R^2^ = 0.972), confirming the reliability of the model, while the adjusted R^2^ (0.924) further indicates excellent agreement between predicted and experimental responses. Overall, these results validate the adequacy of the quadratic polynomial model for describing and predicting melanin production within the studied range of factors.

**Fig. 5:**
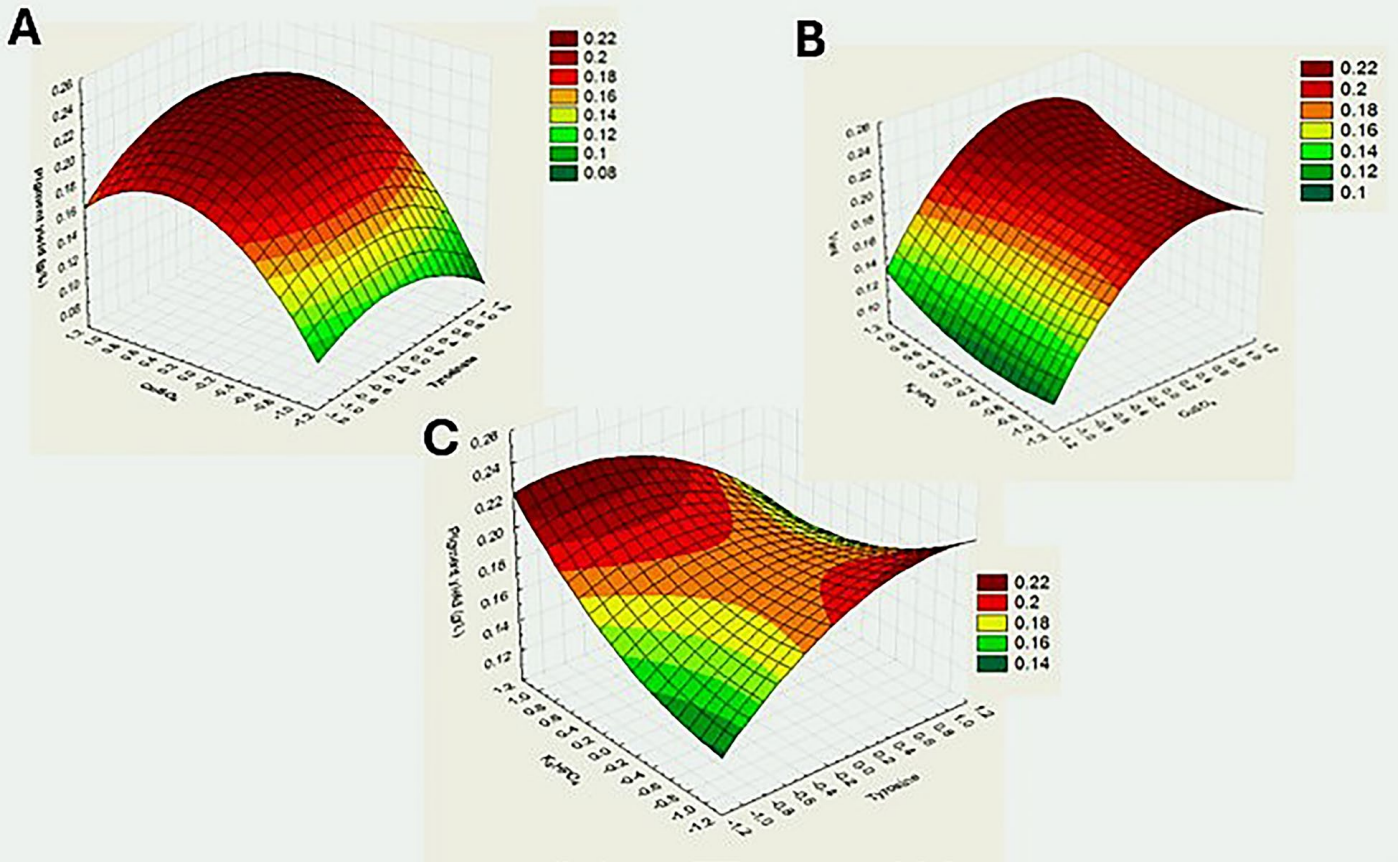
Three-dimensional response surface plots illustrating the effects of the tested variables on melanin production by *Bacillus* sp. EGY7: **A** L-tyrosine (X1) and CuSO_4_ (X2), **B** CuSO_4_ (X2) and K_2_HPO_4_ (X3), and **C** L-tyrosine (X1) and K_2_HPO_4_ (X3)

The interactive effects of the independent variables on pigment yield were further visualized in three-dimensional response surface plots (Fig. 5A–C), which highlight the optimal ranges of L-tyrosine, CuSO_4_, and K_2_HPO_4_ concentrations and reveal the antagonistic interaction between L-tyrosine and K_2_HPO_4_.

The optimal concentrations of the three significant components, estimated from the maximum point of the polynomial model using the SOLVER function in Microsoft Excel, were 0.127 g % L-tyrosine, 0.0162 g % CuSO_4_·5H_2_O, and 0.5 g % K_2_HPO_4_. Accordingly, the optimized production medium composition (g/L) is peptone (20), yeast extract (1), (NH_4_)_2_SO_4_ (0.5), 5.0 g/L K_2_HPO_4_ (5), L-tyrosine (1.27), CuSO_4_·5H_2_O (0.162), and FeSO_4_ (0.5). The calculated melanin yield of *Bacillus* sp. EGY7 in this medium is 0.208 g/L, corresponding to a model accuracy of 86.6%.

It is important to note that the product obtained during the optimization process is authentic melanin rather than a melanoidin derivative. The absence of reducing sugars, the primary reactants involved in the classical Maillard reaction pathway, across all tested optimization conditions strongly indicates that the pigment produced originated from melanin biosynthesis rather than from non-enzymatic browning or charring byproducts (El Hosry et al. 2025). This distinction is further supported by FTIR analysis, which did not reveal the carbohydrate-derived cross-linking bands typically associated with melanoidins. Instead, the spectra exhibited characteristic bands corresponding to the indolic framework of melanin, including aromatic C=C stretching and conjugated quinone C=O vibrations. Collectively, these findings confirm that the recovered pigment is consistent with authentic melanin and not a melanoidin-like byproduct.

### Bacterial melanin-derived carbon dots (MCDs)

Controlled culture conditions, particularly the optimization of medium components, enhanced melanin bioproduction by *Bacillus* sp. EGY7 while preserving its structural integrity. Despite melanin’s abundant aromatic framework and intrinsic heteroatoms, features that make it an excellent precursor for graphitic core formation and in situ heteroatom doping, its application in carbon dot synthesis has been only rarely explored (Khataminejad et al. 2025; Xiao et al. 2017; Shi et al. 2021; Hu et al. 2016), and to the best of our knowledge, not from bacterial sources. This positions the current work as a promising approach to a sustainable, melanin-derived nanoform that preserves the pigment’s intrinsic chemical functionality while imparting bright, tunable PL and excellent nanoscale dispersibility.

A simple one-step hydrothermal carbonization method enabled the transformation of *Bacillus* sp. EGY7-derived melanin into functional MCDs. The hydrothermal conditions (200 °C, 36 h) were selected based on literature reports for melanin-derived carbon dots, including Hu et al. (2016), who employed more severe conditions (220 °C, 48 h) for commercially sourced melanin. Given that the biologically produced melanin used in the present study may differ in purity and macromolecular structure, potentially influencing its carbonization behavior, milder conditions were adopted to ensure sufficient carbonization while preserving surface functional groups that enhance fluorescence and quantum yield.

#### Physical properties

**Fig. 6:**
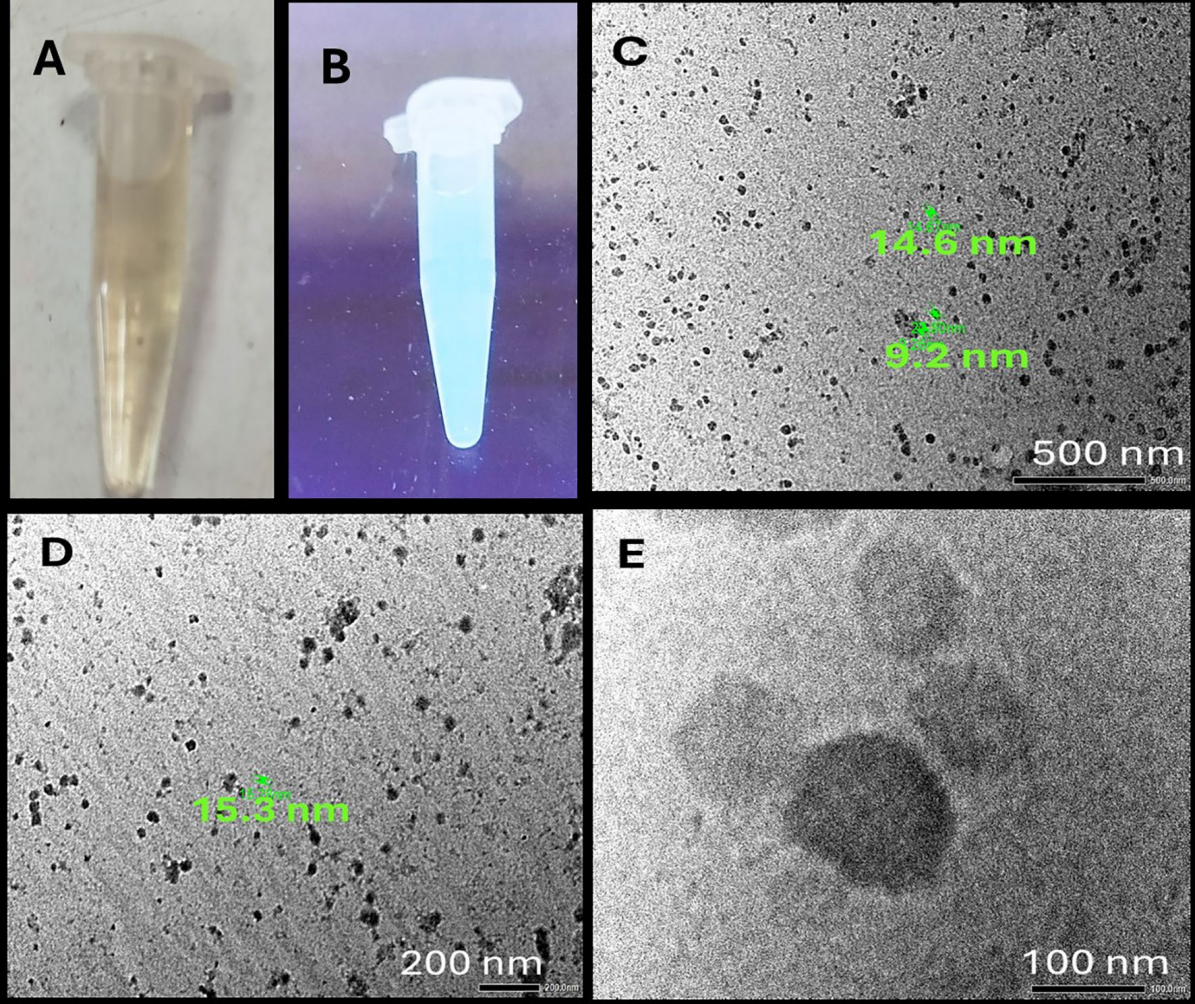
Images of *Bacillus* sp. EGY melanin-produced carbon dots (MCDs). **A** and **B** digital photographs of MCDs dispersed in water showing a clear solution under visible light and strong blue photoluminescence under UV illumination. **C**–**E** TEM micrographs of MCDs at different magnifications, with scale bars of 500, 200, and 100 nm, respectively

Aqueous dispersions of MCDs were optically clear under visible light but displayed intense blue (PL) under UV excitation (Fig. 6A and B), confirming the successful conversion of bacterial melanin into highly fluorescent CDs. The excitation-dependent emission profile, a hallmark of CDs arising from quantum confinement and surface state transitions (Ai et al. 2021), underscores their suitability for tunable optical applications, particularly bioimaging. A zeta potential of –12.8 mV in deionized water indicates electrostatic stabilization to ensure long-term dispersibility in aqueous systems, a critical improvement over native melanin.

TEM analyses (Fig. 6C–E) reveal well-dispersed, quasi-spherical nanoparticles with uniform morphology with no sign of aggregation. The observed particle size of the MCDs (9–15 nm), was slightly larger than the conventional size range typically reported for CDs (< 10 nm). Similar findings have been described for melanin-derived CDs with sizes up to 40–45 nm (Xiao et al. 2017; Hu et al. 2016). Such larger sizes are often observed when natural pigments or biomass are used as precursors, where molecular complexity, incomplete carbonization, and intermolecular crosslinking favor the formation of nanoscale aggregates. Nevertheless, the optical and surface properties confirmed the classification of these nanostructures as MCDs. Taken together, these features highlight MCDs as relatively stable, intrinsically fluorescent nanostructures with strong potential for imaging and therapeutic applications.

#### Stability of melanin carbon dots

The synthesized MCDs exhibited excellent stability across acidic, neutral, and alkaline media, with fluorescence retention values of 97%, 96%, and 94%, respectively after 72 h of incubation at 4°C. Only minor changes in absorbance were observed, while the fluorescence intensity remained largely unchanged, indicating good physicochemical stability over a broad pH range. This high stability aligns with previous reports where surface-passivated CDs showed negligible fluctuation over extended storage in diverse ionic environments (Chen et al. 2023). Photostability studies revealed complete preservation of the initial fluorescence intensity (100% retention) after 72 h of continuous light exposure, demonstrating excellent resistance to photobleaching. This remarkable stability highlights the robustness of the carbon dots and supports their potential use in applications requiring prolonged illumination, such as bioimaging, biosensing, and optoelectronic devices (Liu et al. 2025b).

#### Spectral characteristics of MCDs

**Fig. 7:**
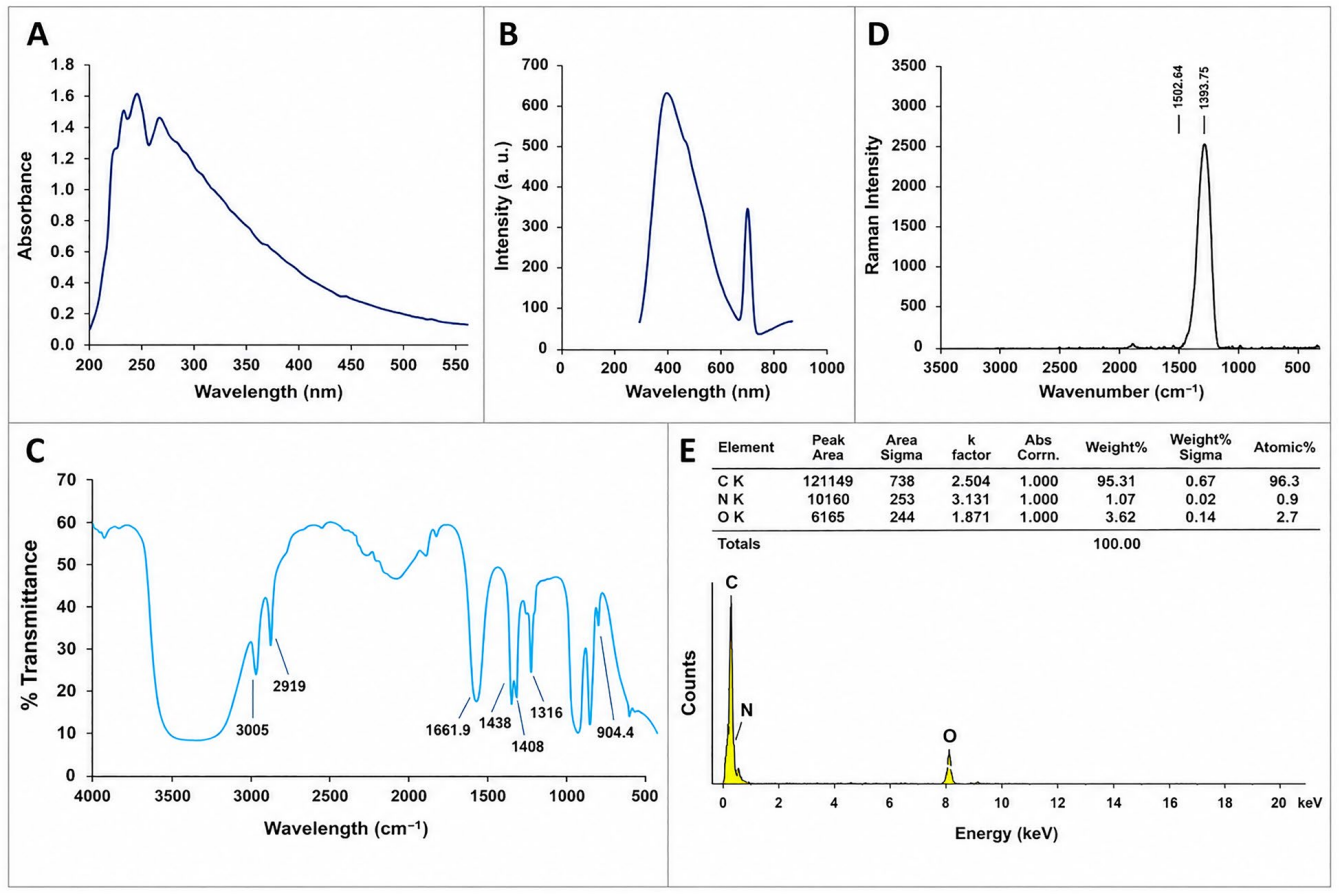
Spectral characteristics of bacterial melanin-derived carbon dots (MCDs). **A** UV–Vis absorption spectrum; **B** Photoluminescence spectrum; **C** FTIR spectrum; **D** Raman spectrum; **E** energy-dispersive X-ray (EDX) spectrum

The UV–Vis spectrum of MCDs exhibits two prominent absorption peaks at 235 and 265 nm (Fig. 7A), consistent with previously reported findings (Alcalá-Alcalá et al. 2023). In CDs, absorption features typically arise from π–π* transitions of aromatic C=C bonds around 230–240 nm and n–π* transitions near 260–270 nm attributed to oxygen-containing groups. Accordingly, the absorption peak at 235 nm can be ascribed to π–π* transitions within aromatic sp^2^-hybridized domains, indicating the presence of conjugated π-electron systems, a hallmark of carbon-based nanomaterials such as CDs and graphene quantum dots (Zhu et al., 2012) . The second peak at 265 nm is attributed to n–π* transitions associated with heteroatom-containing functional groups, including carbonyl, hydroxyl, and amine moieties (Zhu et al., 2012). Collectively, these spectral characteristics suggest that the prepared MCDs possess a structure enriched with conjugated aromatic domains and diverse surface functionalities, consistent with the features of melanin-derived or melanin-mimetic CDs (Hu et al. 2016).

The PL spectrum of MCDs (Fig. 7B) displays two distinct emission maxima at 428.5 nm and 664.5 nm across a broad excitation range of 350–750 nm, consistent with reported findings (Hu et al. 2016). The excitation-dependent fluorescence, with maximum emission extending into the near-infrared region, is particularly advantageous for biological imaging, where deeper tissue penetration and reduced background interference are critical. The calculated QY was 5.05 ± 0.31%, placing MCDs produced in this study among luminescent biogenic carbon dots. The relatively low QY of MCDs derived from melanin produced by the marine-associated *Bacillus* sp. EGY7, compared with many other biogenic carbon dots, is likely attributable to the intrinsic heterogeneous structure of melanin and the nature of the precursor itself. Carbon dots derived from various biomass sources often exhibit higher QYs due to the presence of naturally occurring heteroatoms, such as nitrogen, phosphorus, and sulphur, originating from amino acids, proteins, and carbohydrates. These heteroatoms can be incorporated into the carbon framework during synthesis, creating surface and electronic states that enhance radiative recombination and fluorescence efficiency (Xiang And Tan 2022).

The FTIR spectrum of MCDs (Fig. 7C) displays characteristic absorption bands at 3387.5, 3005.5, 2919.9, 1661.9, 1438, 1408.3, 1019.7, 904.4, and 706.8 cm^−1^. The broad band at 3387.5 cm^−1^ corresponds to O–H stretching, while the absorption at 2919.9 cm^−1^ is attributed to aliphatic C–H stretching. The peak at 1661.9 cm^−1^ confirms C=O/C–N stretching of amide or amine groups, and the band at 3005.5 cm^−1^, along with those at 1438 and 1408.3 cm^−1^, represent aromatic C=C and=CH vibrations of the benzene ring. The absorption at 1019.7 cm^−1^ is assigned to C–O stretching of primary alcohols, whereas the bands at 904.4 and 706.8 cm^−1^ are associated with aromatic C–H bending. Collectively, the FTIR profile indicates that the MCDs retain abundant CN and polycyclic aromatic structures, in agreement with previous reports (Hu et al. 2016).

Raman spectroscopy was further employed to probe the internal carbon structure of MCDs, providing complementary insights into the graphitization degree and defect states. The Raman spectrum of MCDs (Fig. 7D) exhibited two characteristic peaks at 1502.64 cm^−1^ and 1369.75 cm^−1^ which can be attributed to vibrational modes commonly observed in melanin, albeit with slight downshifts compared to the standard ~ 1580 cm^−1^ and ~ 1380 cm^−1^ bands (Huang et al. 2004)**.** The peak at 1502.64 cm^−1^ is assigned to aromatic ring stretching vibrations, analogous to the G band, while the peak at 1369.75 cm^−1^ corresponds to C–C stretching in aromatic systems and possible C–H bending, consistent with the D band. Notably, the D band (1369.75 cm^−1^) exhibited higher intensity than the G band (1502.64 cm^−1^), suggesting a higher degree of disorder, defects, and sp^2^-hybridized carbon domains within the MCDs. This prominent D/G intensity ratio is characteristic of CDs and aligns with the structural features of melanin-derived carbonaceous materials.

EDX analysis confirmed that MCDs are predominantly composed of carbon (95.31 wt %, 96.33 at %) with minor contributions from oxygen (3.62 wt %, 2.75 at %) and trace nitrogen (< 1 at %) (Fig. 7E). The high carbon content reflects the graphitic backbone of the CDs, while the presence of oxygen- and nitrogen-containing atoms indicates surface functionalities that enhance dispersibility and provide potential active sites for bioconjugation.

**Fig. 8:**
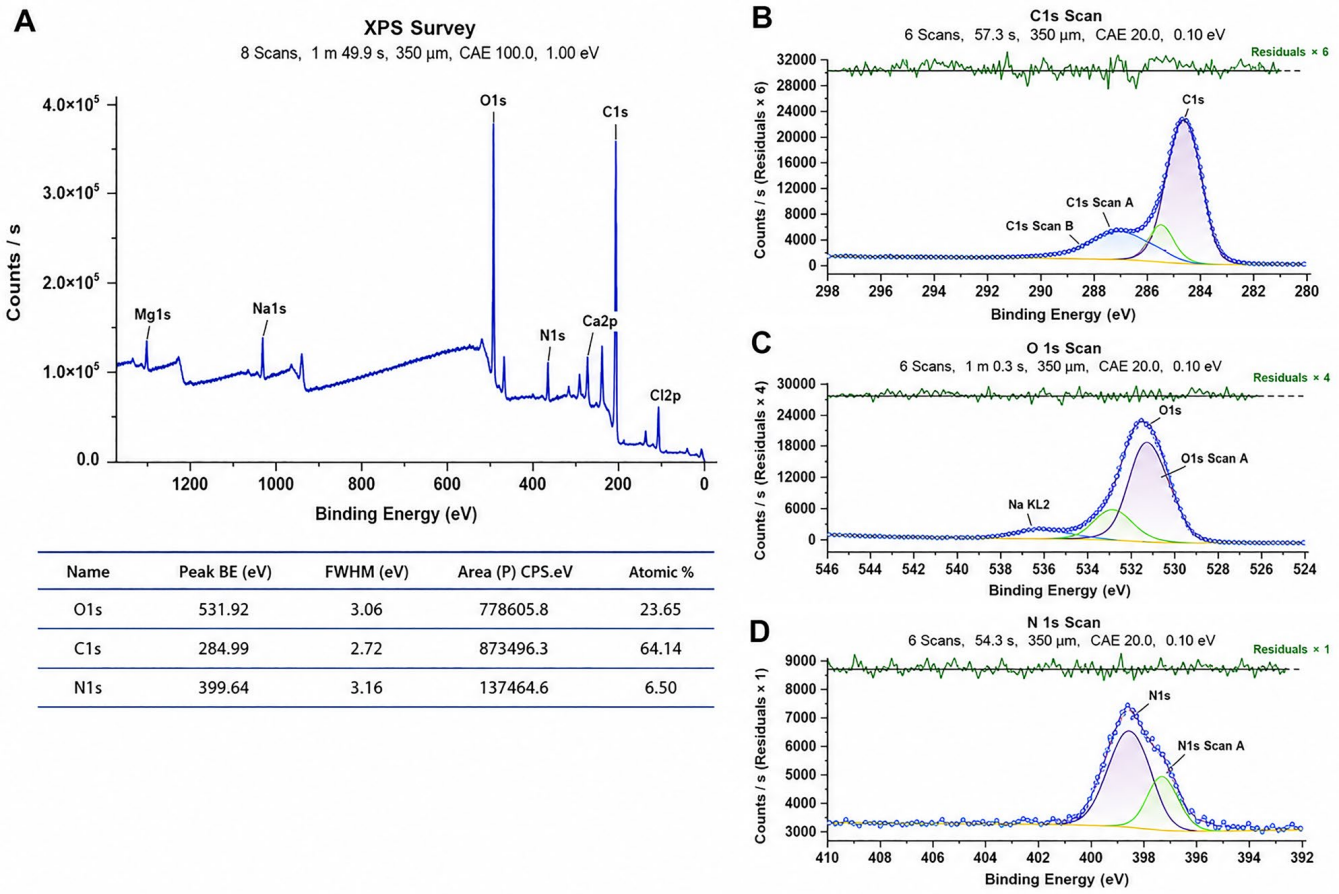
Detailed X-ray photoelectron spectroscopy (XPS) analysis including **A** survey spectrum showing the main C, N, and O peaks, **B** high-resolution C1s spectrum deconvoluted into C–N/C–O, C═N, and C═O groups (57.3 s, 350µm, CAE 20.0, 0.10 eV), **C** high-resolution O1s spectrum indicating C═O and C–OH/C–O–C groups (1m0.3 s, 350µm, CAE 20.0, 0.10 eV), and **D** high-resolution N1s spectrum showing N–C/N–H and C–N–C functionalities ( 54.3 s, 350µm, CAE 20.0, 0.10 eV)

X-ray photoelectron spectroscopy (XPS) analysis was carried out to investigate the surface chemical composition of MCDs. The survey spectrum (Fig. 8A) displayed three main peaks at 284.9, 399.6, and 531.9 eV, corresponding to C1s, N1s, and O1s, respectively. Deconvolution of the C1s spectrum (Fig. 8B) revealed three peaks at 284.51, 285.97, and 287.57 eV, attributed to C–N/C–O, C=N, and C=O groups, respectively (Lei et al. 2018)**.** The N1s spectrum (Fig. 8C) exhibited two peaks at 398.23 and 399.72 eV, assigned to C–N–C and N–C/N–H functionalities. Similarly, the O1s spectrum (Fig. 8D) showed two peaks at 531.17 and 532.52 eV, corresponding to C=O and C–OH/C–O–C groups (Hu et al. 2016). Collectively, these results confirm the presence of abundant oxygen‑ and nitrogen‑containing functional groups on the MCD surface, highlighting their heteroatom‑doped nature.

The C/N ratio determined from the XPS atomic percentages was approximately 9.9 (C=64.4% and N = 6.5%). This value is consistent with reported compositions of eumelanin-derived materials originating from indolic precursors. Since eumelanin is primarily composed of indole-based units, such as 5,6-dihydroxyindole (DHI) and 5,6-dihydroxyindole-2-carboxylic acid (DHICA), the retention of nitrogen in the carbon dots and the resulting C/N ratio suggest that nitrogen-containing aromatic motifs were preserved during hydrothermal carbonization (Pralea et al. 2019).

Overall, the characterization data verify the successful formation of MCDs under the applied conditions (200 °C and 48 h). The synthesized MCDs showed distinct optical features, structural integrity, and a surface negative charge. In addition, the FTIR, Raman, and XPS analyses reveal abundant oxygen- and nitrogen-containing groups that improve dispersibility and provide active sites for bioconjugation. Despite the moderate QY, the applied hydrothermal conditions were suitable for CD synthesis, enabling the formation of a graphitic core while preserving oxygen- and nitrogen-containing surface functional groups. Hydrothermal parameters, particularly temperature and reaction time, strongly influence nucleation, graphitization, particle growth, and surface passivation. Insufficient carbonization can occur at low temperatures or short reaction times, whereas excessive conditions may promote over-carbonization, aggregation, and larger particle sizes, reducing surface passivation and consequently lowering the QY (Santos et al. 2025).

#### Biological activity of MCDs

Before screening the biological activity of MCDs, in silico analyses were conducted to evaluate the drug-likeness, pharmacokinetics, and predicted bioactivity of melanin pigment. Drug-likeness was assessed using the SMILES notation of melanin (PubChem CID: 6,325,610), along with its chemical structure (Fig. S4A) and molecular formula (C_18_H_10_N_2_O_4_), across multiple computational platforms, including the SwissADME bioavailability radar (Fig. S4B). This evaluation incorporated key physicochemical descriptors including lipophilicity, molecular size, polarity, solubility, saturation, and flexibility**.** Melanin exhibited a molecular weight below 500 Da, consistent with previous reports, supporting its favorable drug-like profile. This was reflected in its drug-likeness as predicted by MolSoft tool (Fig. S4C). However, its negative skin permeability coefficient (log Kp = − 9.11 cm/s) indicates limited dermal penetration likely due to its hydrophobic and structurally complex nature. Overall, melanin demonstrates promising drug-like characteristics, though its restricted skin permeability highlights the need for optimization strategies. Approaches such as melanin-like nanoparticles and microneedle-assisted delivery may enhance transdermal application, particularly in treating hypopigmentation disorders such as vitiligo (Jiang et al. 2025).

The more negative log Kp value the lower the ability of a bioactive compound to penetrate the skin (Table 6). Collectively, melanin alone is predicted to have drug-like properties though with these can be improved.

**Table 6:** Physicochemical properties and predicted biological activities of melanin.

Parameter	Value
SwissADME parameters	
Molecular weight	318.28 g/mol
Log S (SILICOS−IT) Solubility	−7.05 2.84 × 10⁻^5^ mg/mL (8.93 × 10⁻^8^ mol/L)
Class	Poorly soluble
Lipophilicity Log P_o/w_ (SILICOS-IT)	5.42 (lipophilic)

Pa	Pi	Activity
PASS-predicted biological activities (Pa: probability of activity; Pi: probability of inhibition)		
0.808	0.011	Antineoplastic
0.209	0.050	Antioxidant
0.164	0.039	Antibacterial (ophthalmic)
0.221	0.100	Antibacterial
0.049	0.048	Antifungal (Pneumocystis)

Consequently, the PASS Online tool was employed to predict the biological activity spectrum of melanin. This algorithm considers a compound bioactive if the probability of activation (Pa) exceeds the probability of inhibition (Pi).

The analysis indicated that melanin has a high probability of antineoplastic activity, moderate antioxidant potential, and low antimicrobial activity, with the latter reflected by Pa and Pi values being comparable (Table 6). These in silico predictions guided the subsequent in vitro evaluation of the antimicrobial, antioxidant, and antineoplastic activities of MCDs.

#### Antimicrobial activity

The antimicrobial activity of MCDs evaluated against *E. coli*, *S. aureus*, and *C. albicans* using the well diffusion method, revealed limited efficacy. Among the tested microorganisms, only *E. coli* showed modest susceptibility, even at the highest concentration tested (5 mg/mL), with a maximum inhibition zone of 8 mm. It is well established that the antimicrobial mechanisms of CDs involve both physical and chemical interactions with microbial membranes, including surface adhesion, wrapping, and membrane disruption (Liang et al. 2021). Accordingly, their antimicrobial efficacy is strongly influenced by physicochemical properties such as particle size and size distribution (Sun et al. 2021), surface charge (Hu et al. 2016) as well as surface functional groups and heteroatom doping (Guo et al. 2022; Romulo et al. 2025). In the present study, the relatively larger particle size of MCDs (9–15 nm), their negative zeta potential (− 18 mV), and the low heteroatom content indicated by EDX analysis likely contribute to the weak antimicrobial activity observed.

#### Antioxidant activity

In the DPPH assay, MCDs (5 mg/mL) showed 20% radical-scavenging activity after 2 h, lower than values reported for heteroatom-doped or surface-functionalized carbon dots (Pan et al. 2026). The incorporation of heteroatoms such as sulphur, nitrogen and phosphorus modifies the electronic structure of the carbon lattice and introduces additional functional groups (Hussain et al. 2025). These heteroatom-containing sites stabilize the reactive oxygen species and the free radicals through donating hydrogen atoms. In addition, their ratio controls how easy the carbon dot can donate electrons for radicals quenching, thereby improving their antioxidant properties (Kasif et al. 2024). Nonetheless, the intrinsic melanin-derived structure of MCDs featuring oxygenated surface groups, defect sites, conjugated π-domains, and quinone/hydroquinone redox couples, contributes additional intrinsic redox activity that partially compensates for lack of heteroatom modification, consistent with reports on non-doped carbonaceous nanomaterials (Ji et al. 2019; Sharma et al. 2026).

The particle size, in terms of S/V, is another factor along with the heteroatom ratio controlling the antioxidant potency of CDs. In relatively small particles, the surface defects and functional group are significantly exposed on the surface, allowing better electron transfer and consequently higher radical scavenging efficiency (Wang et al. 2025). The prepared MCDs possess a modest S/V ratio (0.4–0.67nm^−1^) based on particle diameter range of 9–15 nm, which might contribute to the low antioxidant efficiency. In a previous study (Wibowo et al. 2026), the carbon dots (CDs) were synthesized in various sizes to evaluate size-dependent biological activity; among these, the smallest CDs (2.16–4.38 nm) showed the highest antioxidant efficiency. This enhanced performance is attributed to the fact that smaller particle sizes increase the surface-area-to-volume ratio, thereby exposing a greater density of active surface functional groups.

#### Cytotoxicity and cytocompatibility

The cytotoxicity and cytocompatibility of native melanin and MCDs were comparatively evaluated using MCF-7 and MDA-MB-231 breast cancer cell lines along with human skin fibroblasts (HSFs) as a normal cell model. Breast cancer cell lines were selected to evaluate the anticancer activity of MCDs because melanin has previously demonstrated activity against breast cancer cells, with enhanced efficacy reported for melanin-based nanoparticles (Rajabathar et al. 2023; Guo et al. 2023). Moreover, despite the improved dispersibility, stability, and bioactivity of the MCDs developed in this study, their anticancer potential remains to be explored. Therefore, two cell lines, MCF-7 and MDA-MB-231 were chosen because they represent two distinct breast cancer subtypes: MCF-7 is ER + /PR + (hormone-sensitive), while MDA-MB-231 is triple-negative (ER − /PR − /HER2 −), highly aggressive, and metastatic. Testing against both cell lines allows to evaluate whether MCDs in this study have broad-spectrum antitumor activity across subtypes or subtype-specific efficacy. This pairing is particularly important because triple-negative breast cancer lacks targeted therapies, making it a critical model for novel anticancer agents. Additionally, this cell line combination is widely established in breast cancer literature, enabling meaningful comparison with published data.

**Fig. 9:**
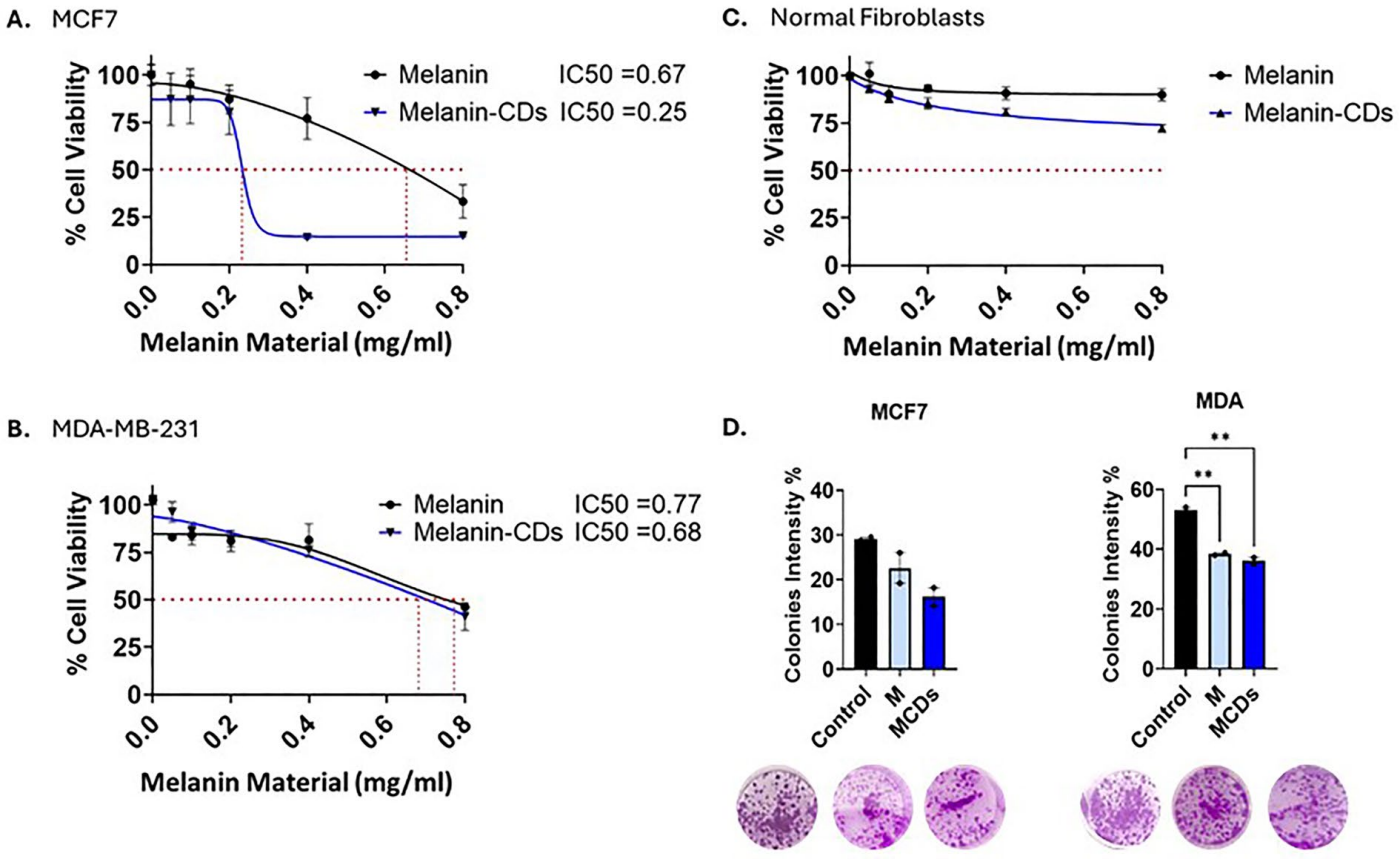
Cytotoxicity and cytocompatibility of native melanin and melanin carbon dots (MCDs). Cell viability MTT assay on **A** MCF7 cells, **B** MDA-MB-231 cells and **C** human skin fibroblasts (HSFs) using different concentrations of melanin (black line, n = 5) and MCDs (blue line, n = 5). **D** Colony forming assay on MCF7 cells and MDA-MB-231 cells (n = 3). Representative colony images (bottom) corroborate these findings. Statistical significance is indicated (**p < 0.01)

*Cytotoxicity against cancer cells* As shown in Fig. 9A,B, native melanin displayed moderate cytotoxicity against breast cancer cells (IC_50_: 0.67 mg/mL for MCF7 and 0.77 mg/mL for MDA-MB-231). MCD conversion boosted potency (IC_50_: 0.25 mg/mL for MCF7; 0.68 mg/mL for MDA-MB-231), likely via enhanced endocytic uptake in cancer cells. IC_50_ 0.67 0.77. Conversion into MCDs enhanced anticancer activity, reducing the IC_50_ values to 0.25 mg/mL and 0.68 mg/mL, respectively. This improvement may be attributed to the nanoscale size of MCDs, which facilitates cellular internalization through endocytic pathways. Although the MCDs possessed a negative surface charge, nanoparticle uptake is not solely governed by zeta potential. In physiological media, protein corona formation can alter nanoparticle surface characteristics and promote cellular recognition, enabling efficient uptake through both non-specific and receptor-mediated endocytosis (Fiori et al. 2022; Öztürk et al. 2024). MCF7’s greater sensitivity reflects ROS-induced DNA damage (Şimşek et al. 2020). While MDA-MB-231’s lower response aligns with TNBC’s pro-survival pathways (Grubczak et al. 2021).

*Cytocompatibility with normal cells* Both native melanin and MCDs showed low toxicity to normal HSFs (> 70% viability; IC_50_ ≈4.9 mg/mL; Fig. 9C), yielding high selectivity indices (MCDs: 19.6 vs. MCF7; 7.2 vs. MDA-MB-231). This selectivity arises from fibroblasts’ low endocytic uptake, reduced membrane dynamics, and robust antioxidant defences, e.g. catalase and glutathione, that neutralize nanoparticle-induced ROS (Bromma et al. 2020). CDs typically spare healthy cells at therapeutic doses due to endocytosis and proliferation dependence (Kumar et al. 2026).

*Long-term antiproliferative effects* In the colony formation assay, 7-day treatment showed that MCDs more potently inhibited MCF7 and MDA-MB-231 colony formation than native melanin (Fig. 9D), especially in MDA-MB-231. MCDs’ nanoscale properties exploit TNBC’s high macropinocytosis/endocytosis, generating ROS that disrupt mitochondria and induce apoptosis in ROS-sensitive TNBC unlike MCF7 (Grubczak et al. 2021), explaining MDA-MB-231’s short-term resistance but long-term vulnerability. (verified by images, Fig. 9D bottom). Other studies have reported differences in response to long-term drug treatment attributing these differences to the distinct molecular and mutational signature of the two cell lines (Mahmoud et al. 2023). Their genotypic and phenotypic differences are manifested on different levels exceeding their differences in hormone receptor expression. Metabolically, MDA-MB-231 cells . In addition, MCF7 are rather epithelial whereas MDA-MB-231 are mesenchymal (Theodossiou et al. 2019). A previous study by Jia et al. (2014) referred the difference of susceptibilities of both cell lines to different drugs. In general, MDA-MB-231 cells tend to form smaller and less dense colonies than MCF7 cells whose cells are rather compact and dense. Again, this can be attributed to the mesenchymal like nature of MDA-MB-231 cells.

#### Molecular target prediction for melanin

**Fig. 10:**
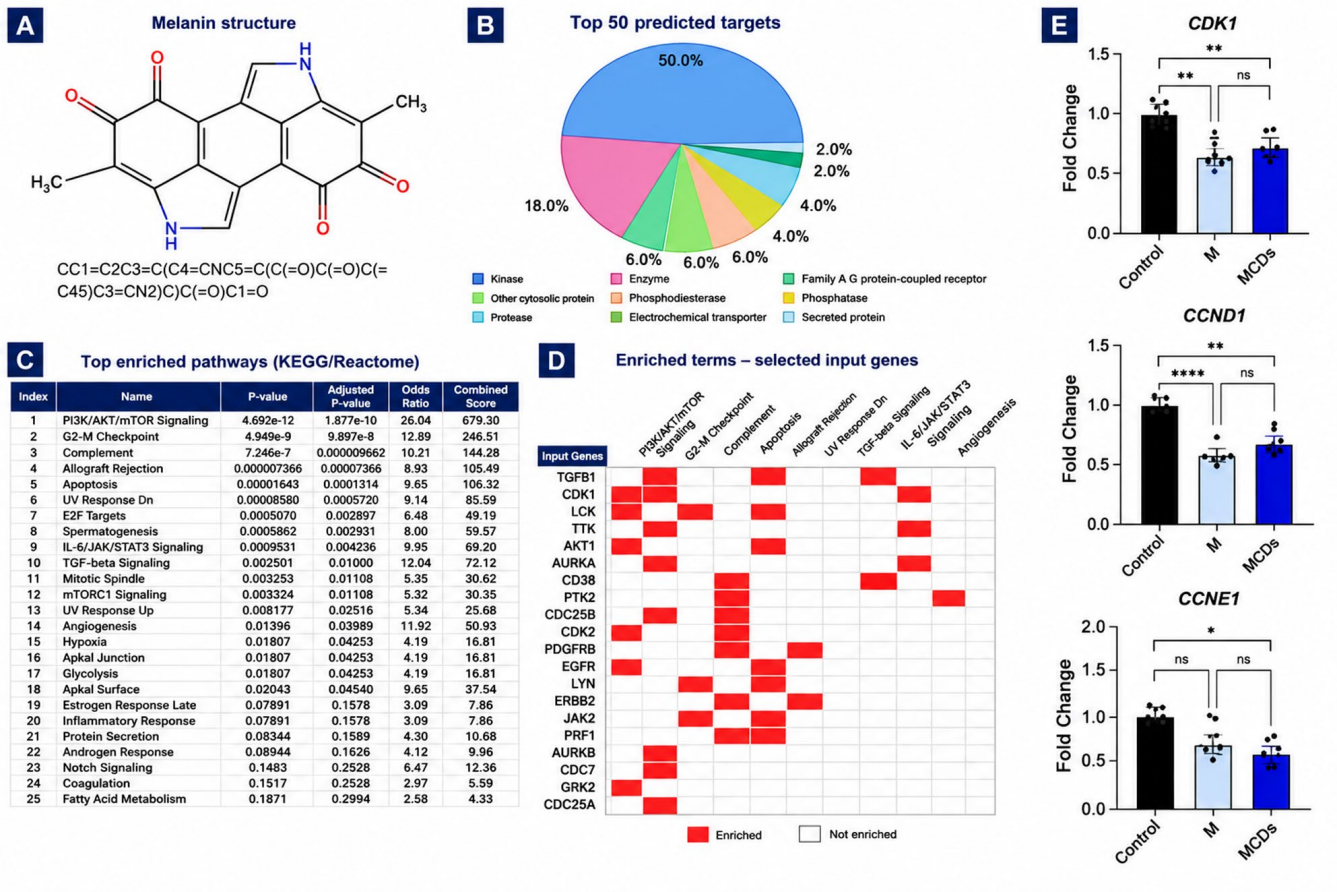
In silico analysis of melanin molecular targets. **A** Predicted molecular targets based on melanin structure highlighting the molecular families of the top 50 candidates. Gene set enrichment analysis showing **B** Enriched pathways for the predicted molecular targets sorted by increasing p-value and **C**, **D** Clustergram of the top 10 predicted genes and their involvement in signaling pathways as predicted using MSigDB Hallmark 2020 database. **E** Expression profile by qPCR of MCF7 after treatment with 0.25 mg/ml melanin (M) or melanin carbon dots (MCDs). Cells were treated for 24 h (n = 3)

Following the evaluation of the biological activity of native melanin and MCDs in cancer cells using functional assays, it was important to gain deeper insight into the underlying mechanisms responsible for their cytotoxic effects. Using the SMILES sequence and structural information of melanin retrieved from PubChem (Fig. S4A), its potential molecular targets were predicted. These targets were predominantly kinases (50%, Fig. 10A), along with other protein classes such as proteases, phosphodiesterases, and G protein-coupled receptors, all of which play critical roles in regulating cell growth and survival.

Gene set enrichment analysis revealed that the predicted targets were enriched in key regulatory pathways, including those involved in cell growth and proliferation, such as the PI3K/AKT/mTOR signaling pathway and the G2/M checkpoint. Additionally, pathways associated with apoptosis and inflammation, such as TGF-β signaling and IL-6/JAK/STAT3 signaling, were also significantly enriched (Fig. 10B). Notably, several key targets identified, including TGFB1, CDK1, and AKT1, are well-established regulators implicated in multiple cancer types (Fig. 10C, D).

To confirm the in silico results of target prediction, the expression of cell cycle markers like *CDK1*, *CCND1* and *CNNE1* was measured. The qPCR results demonstrated that treatment with MCDs significantly downregulated the expression of *CDK1, CCND1*, and *CCNE1* in MCF7 breast cancer cell line (Fig. 10E). These genes play interconnected roles in cell-cycle regulation and cellular proliferation.

CCND1 (Cyclin D1) and CCNE1 (Cyclin E1) are key regulators of G1-phase progression and the G1/S transition. Cyclin D1 forms complexes with CDK4/6 to initiate cell-cycle progression, while Cyclin E1 promotes entry into the S phase through activation of CDK2. Dysregulation and overexpression of both CCND1 and CCNE1 are frequently associated with uncontrolled proliferation in breast cancer cells.

CDK1 is a master cell-cycle kinase that governs cell-cycle progression, particularly the G2/M transition, and can compensate for the activity of other cyclin-dependent kinases under certain conditions. Consequently, suppression of CDK1 expression can disrupt cell-cycle progression and ultimately inhibit cellular proliferation.

Regulation of cell cycle is orchestrated by cyclin-dependent kinases as CDK1 and other regulators such as cyclin D1 and cyclin E1. Because CCND1 and CCNE1 drive G1/S progression while CDK1 is essential for completion of the cell cycle and mitotic entry, their simultaneous downregulation suggests a coordinated suppression of cell-cycle progression, providing a mechanistic explanation for the observed reduction in breast cancer cell proliferation. In concordance with our work, several studies have reported the beneficial outcomes on targeting CDK1 in breast cancer and other cancer types (Ghafouri-Fard et al. 2022).

In addition, melanin is well recognized for its role in maintaining cellular redox balance, as increased melanin synthesis has been associated with enhanced protection against environmental and therapy-induced stressors (Hong et al. 2026). Its ability to scavenge reactive oxygen species (ROS) likely contributes to a more reduced intracellular environment, thereby mitigating oxidative DNA damage and influencing cell survival (Zhu et al. 2022). In silico analysis of predicted melanin targets confirmed these observations and further suggested additional mechanistic pathways through which melanin may exert cytotoxic effects.

## Conclusion

This study establishes marine-associated *Bacillus* sp. EGY7 as a sustainable and scalable source of melanin, offering a green alternative to conventional methods of the pigment production. Conversion of melanin into carbon dots (MCDs) markedly improved dispersibility and bioactivity. The resulting MCDs exhibited excellent cytocompatibility together with selective anticancer activity against breast cancer cells, evidenced by reduced IC_50_ values, pronounced inhibition of colony formation, and high selectivity indices. Complementary in silico analyses revealed potential multi‑target interactions, supporting a multimodal mechanism of action. Collectively, these findings highlight microbial melanin as a renewable precursor for value‑added nanomaterials and position the integration of microbial fermentation with nanoengineering as a sustainable platform for advanced biomedical applications. Future work should focus on targeted functionalization and optimized formulation strategies to further enhance selective anticancer efficacy and other bioactivities of MCDs to broaden their biomedical applications.

## Supplementary Information

Additional file1 (DOCX 2420 KB)

## Data Availability

Data available on request from the authors.
