# Supplementary material for: Optimized melanin production from marine-associated Bacillus sp. EGY7 as a sustainable precursor of carbon dots for biomedical applications
Source: Bioresour Bioprocess. 2026 Jul 27;13(1):106. doi: 10.1186/s40643-026-01100-w (PMC13407830; doi:10.1186/s40643-026-01100-w)
Supplement: Supplementary file 1 — Additional file1 (DOCX 2420 KB) [file 40643_2026_1100_MOESM1_ESM.docx]

**Optimized Melanin Production from Marine-Associated *Bacillus* sp. EGY7 as a sustainable precursor of Carbon Dots for Biomedical Applications**

Hadeer Ali^1^, Labiba El-Kohordagui^2^, Ahmed Hussein^1^, Sherif Hammad^3, 4^, Nefertiti El‑Nikhely^1,5^, Nehad Noby^1*^

^1^ Biotechnology Department, Institute of Graduate Studies and Research, Alexandria University, Egypt.

^2^ Department of Pharmaceutics, Faculty of Pharmacy, Alexandria University, Egypt.

^3^ Pharmaceutical Chemistry Department, Faculty of Pharmacy, Capital University (Formerly Helwan University), Cairo 11795, Egypt.

^4^ Medicinal chemistry Department, Faculty of Pharmacy, Egypt-Japan University of Science and Technology (E-JUST), New Borg El-Arab City, 21934 Alexandria, Egypt.

^5^ Molecular Biotechnology Program, Alamein International University, New Alamein, Egypt.

**Table S1.** Composition of the six media formulations used in the one-variable-at-a-time (OVAT) experiments

| **Component**  **(g L⁻¹)** | **Medium 1**  **(Basal)** | **Medium 2** | **Medium 3** | **Medium 4** | **Medium 5** | **Medium 6** |
| --- | --- | --- | --- | --- | --- | --- |
| Peptone | 20.0 | 20.0 | 20.0 | 20.0 | 20.0 | 20.0 |
| Yeast extract | 1.0 | 1.0 | 1.0 | 1.0 | 1.0 | 1.0 |
| (NH₄)₂SO₄ | 0.5 | 0.5 | 0.5 | 0.5 | 0.5 | 0.5 |
| K₂HPO₄ | 1.0 | 1.0 | 1.0 | 1.0 | 1.0 | – |
| Na₂S₂O₃ | 0.08 | 0.08 | 0.08 | 0.08 | – | 0.08 |
| Fe₂SO₄.5H_2_O | 0.5 | 0.5 | 0.5 | 0.5 | 0.5 | 0.5 |
| L-tyrosine | – | 0.1 | – | _ | _ | _ |
| CuSO₄ | – | – | 0.04 | – | _ | _ |
| MgSO₄ | – | – | – | 0.04 | _ | _ |

**Table S2:** Box Bhnken design (BBD) matrix of three independent variables with their coded and real value

| Trial number | L-tyrosine % (w/v)  (X1) | CuSO4 % (w/v)  (X2) | K_2_HPO_4_ % (w/v)  (X3) |
| --- | --- | --- | --- |
| 1 | 0 (0.15) | 0 (0.015) | 0 (0.3) |
| 2 | 0 (0.15) | 0 (0.015) | 0 (0.3) |
| 3 | 0 (0.15) | -1 (0.005) | 1 (0.5) |
| 4 | 0 (0.15) | 1 (0.025) | -1 (0.1) |
| 5 | -1 (0.05) | -1 (0.005) | 0 (0.3) |
| 6 | 0 (0.15) | 0 (0.015) | 0 (0.3) |
| 7 | 1 (0.25) | 0 (0.015) | -1 (0.1) |
| 8 | 0 (0.15) | 1 (0.025) | 1 (0.5) |
| 9 | 0 (0.15) | -1 (0.005) | -1 (0.1) |
| 10 | -1 (0.05) | 0 (0.015) | 1 (0.5) |
| 11 | -1 (0.05) | 0 (0.015) | -1 (0.1) |
| 12 | 1 (0.25) | 0 (0.015) | 1 (0.5) |
| 13 | 1 (0.25) | 1 (0.025) | 0 (0.3) |
| 14 | 1 (0.25) | -1(0.005) | 0 (0.3) |
| 15 | -1 (0.05) | 1 (0.025) | 0 (0.3) |


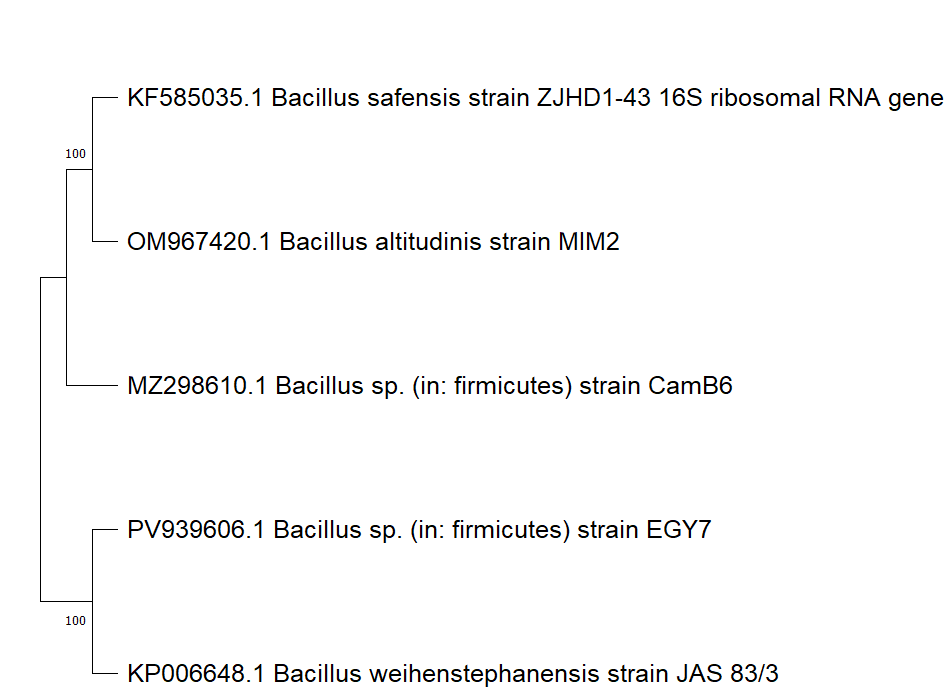


**Fig. S1:** Phylogenetic relationship of EGY strain and other known melanin-producing *Bacillus* strains


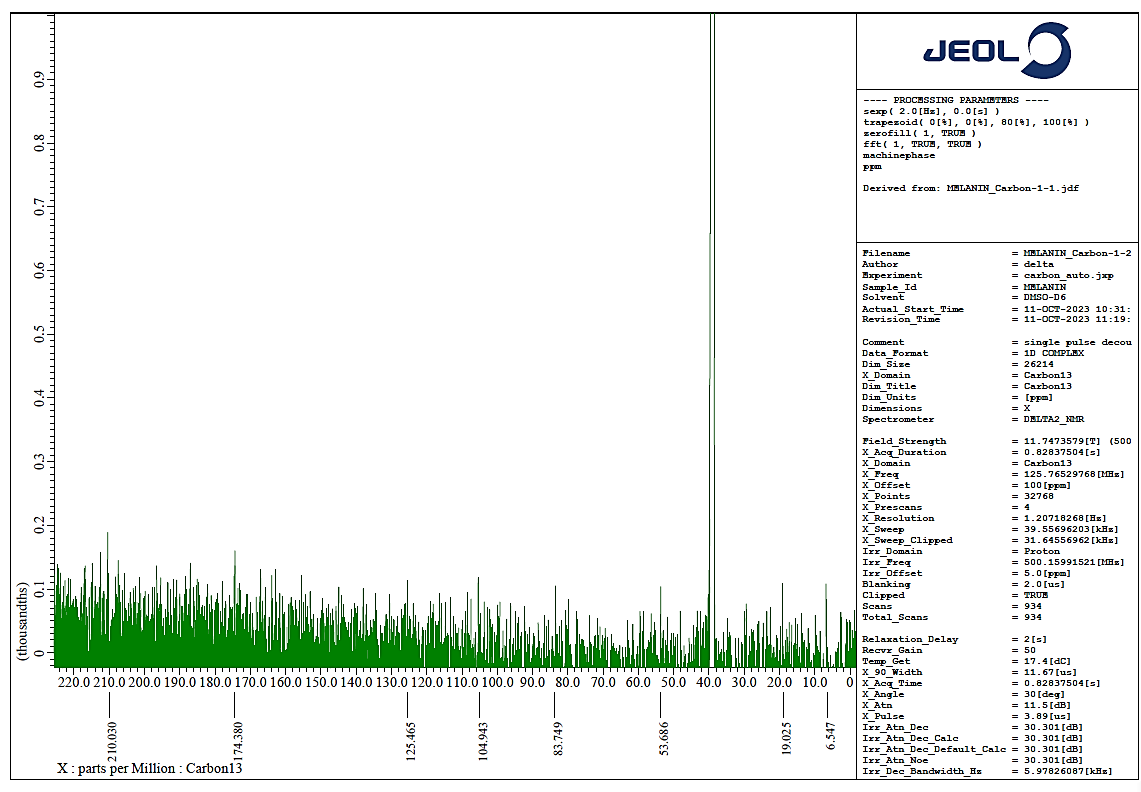


**Fig. S2:** ^13^ C NMR analysis of purified Bacillus sp. EGY7 melanin

The carbon structure of pure bacterial melanin (PBM) was further examined using ^13^C nuclear magnetic resonance (^13^C NMR) spectroscopy. The obtained spectrum showed distinctive signals that correspond to different carbon environments that are frequently present in macromolecular structures like melanin. Notably, a strong resonance was detected at about 177 ppm, which is ascribed to carbonyl carbons (C=O) from ester groups, amides, or carboxylic acids. Because of the oxidative polymerization of phenolic and indolic precursors and the addition of subunits generated from amino acids, these moieties are usually present in melanin [1, 2]. Sp2 hybridized carbons in aromatic systems are indicated by signals in the 100–160 ppm range. These peaks are typical of conjugated aromatic rings, which make up the backbone of polymers of the eumelanin type and are seen in indole, pyrrole, and catechol structures[3]. Extended π-conjugation, a characteristic that distinguishes melanin pigments, is confirmed by these resonances.

Aliphatic carbon atoms connected to nitrogen or oxygen (such as –CH₂–NH– or –CH₂–OH) are usually linked to the signal centered at 56 ppm [4]. Aliphatic -CH₃ and -CH₂- groups are probably the cause of the minor signals that show up between 10 and 40 ppm, indicating the presence of short alkyl chains. The identity of PBM as a heterogeneous, highly aromatic polymer loaded with functional groups including carbonyls, hydroxyls, and amines is supported by the ^13^C NMR spectrum data taken together. These results support the idea that PBM is a eumelanin-like pigment made by microbial metabolic processes and are consistent with previous reports on natural and synthetic melanin [5, 6].


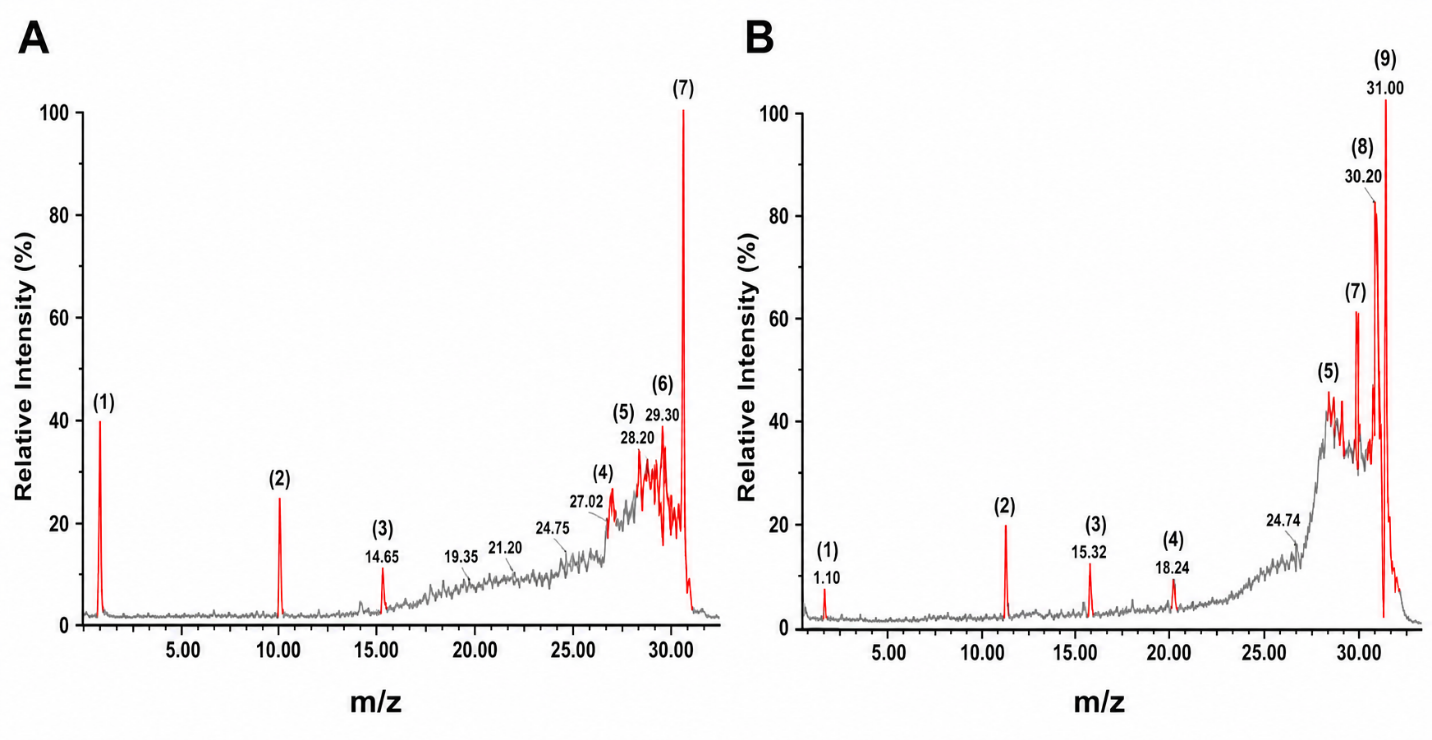
**Fig S3:** Electrospray ionization mass spectrometry (ESI-MS) spectra of purified *Bacillus*-derived melanin: (A) negative ion mode and (B) positive ion mode.


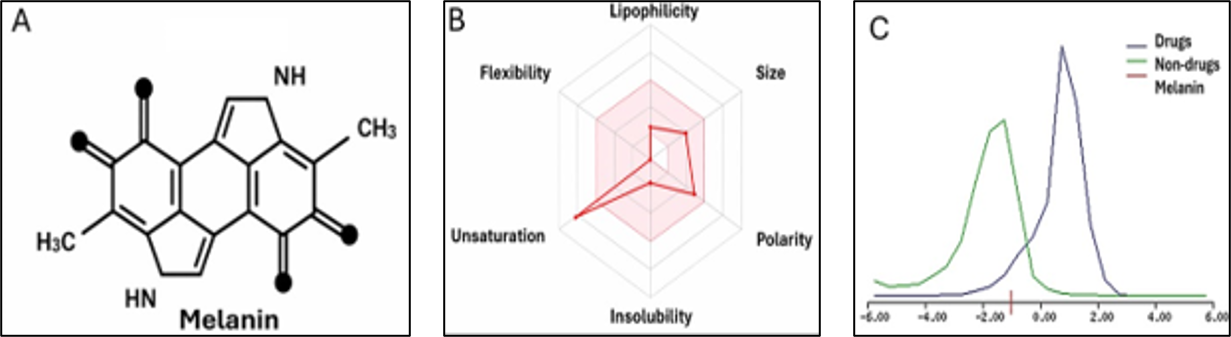


**Fig S4:** *In silico* prediction of the drug-likeness and pharmacokinetic properties of melanin. (A) Molecular structure, (B) bioavailability radar summarizing key physicochemical descriptors relevant to drug-likeness, (C) Skin permeability coefficient.

**References:**


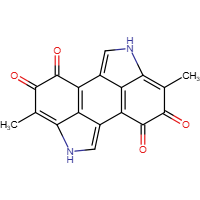


CC1=C2C3=C(C4=CNC5=C(C(=O)C(=O)C(=C45)C3=CN2)C)C(=O)C1=O

A.


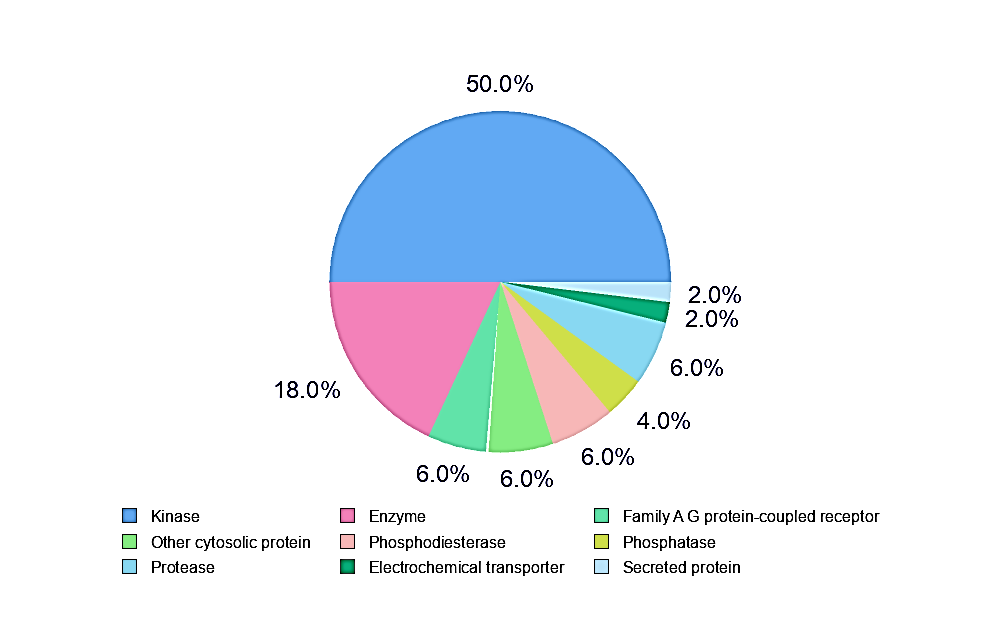


B.

Top 50


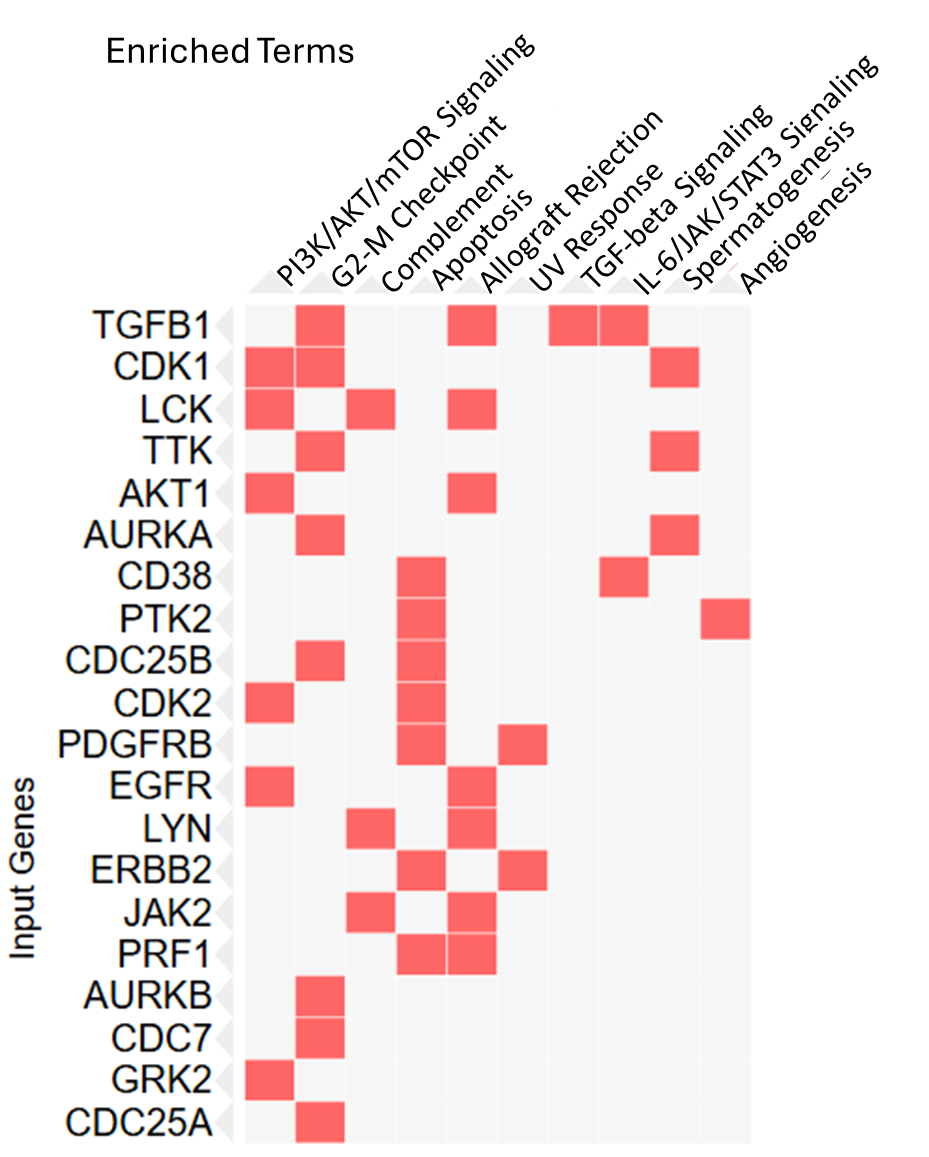

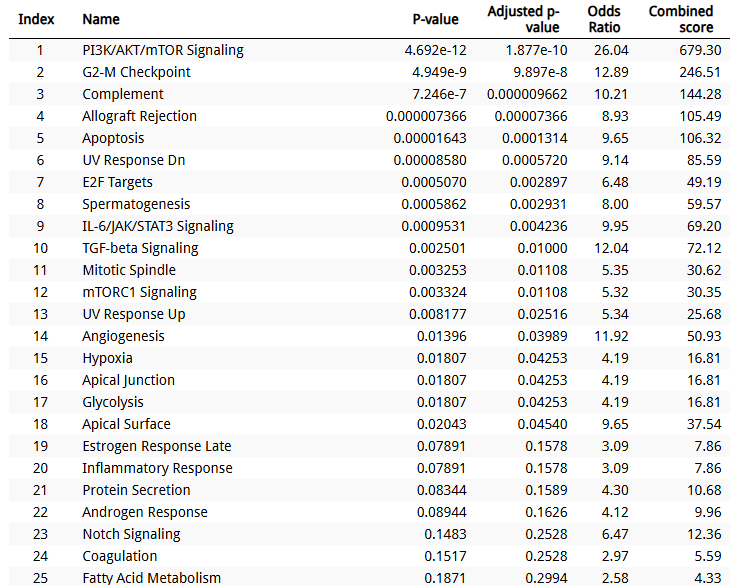


C.

D.

E.

1.d'Ischia, M., et al., *Melanin biopolymers: Tailoring chemical complexity for materials design.* Angewandte Chemie International Edition, 2020. **59**(28): p. 11196-11205.

2.Solano, F., *Photoprotection and skin pigmentation: Melanin-related molecules and some other new agents obtained from natural sources.* Molecules, 2020. **25**(7): p. 1537.

3.Tran-Ly, A.N., et al., *Microbial production of melanin and its various applications.* World Journal of Microbiology and Biotechnology, 2020. **36**: p. 1-9.

4.Mostert, A.B., et al., *Role of semiconductivity and ion transport in the electrical conduction of melanin.* Proceedings of the National Academy of Sciences, 2012. **109**(23): p. 8943-8947.

5.Turick, C.E., et al., *Properties and function of pyomelanin.* Biopolymers, 2010. **449**(72): p. 10.5772.

6.Pralea, I.-E., et al., *From extraction to advanced analytical methods: The challenges of melanin analysis.* International journal of molecular sciences, 2019. **20**(16): p. 3943.
